# *Pseudomonas aeruginosa* Exhibits Frequent Recombination, but Only a Limited Association between Genotype and Ecological Setting

**DOI:** 10.1371/journal.pone.0044199

**Published:** 2012-09-06

**Authors:** Timothy J. Kidd, Stephen R. Ritchie, Kay A. Ramsay, Keith Grimwood, Scott C. Bell, Paul B. Rainey

**Affiliations:** 1 Queensland Paediatric Infectious Diseases Laboratory, Queensland Children’s Medical Research Institute, Royal Children’s Hospital, The University of Queensland, Brisbane, Queensland, Australia; 2 New Zealand Institute for Advanced Study and Allan Wilson Centre for Molecular Ecology and Evolution, Massey University at Albany, Auckland, New Zealand; 3 Department of Thoracic Medicine, The Prince Charles Hospital, Brisbane, Queensland, Australia; 4 Max Planck Institute for Evolutionary Biology, Plön, Germany; University of Edinburgh, United Kingdom

## Abstract

*Pseudomonas aeruginosa* is an opportunistic pathogen and an important cause of infection, particularly amongst cystic fibrosis (CF) patients. While specific strains capable of patient-to-patient transmission are known, many infections appear to be caused by unique and unrelated strains. There is a need to understand the relationship between strains capable of colonising the CF lung and the broader set of *P. aeruginosa* isolates found in natural environments. Here we report the results of a multilocus sequence typing (MLST)-based study designed to understand the genetic diversity and population structure of an extensive regional sample of *P. aeruginosa* isolates from South East Queensland, Australia. The analysis is based on 501 *P. aeruginosa* isolates obtained from environmental, animal and human (CF and non-CF) sources with particular emphasis on isolates from the Lower Brisbane River and isolates from CF patients obtained from the same geographical region. Overall, MLST identified 274 different sequence types, of which 53 were shared between one or more ecological settings. Our analysis revealed a limited association between genotype and environment and evidence of frequent recombination. We also found that genetic diversity of *P. aeruginosa* in Queensland, Australia was indistinguishable from that of the global *P. aeruginosa* population. Several CF strains were encountered frequently in multiple ecological settings; however, the most frequently encountered CF strains were confined to CF patients. Overall, our data confirm a non-clonal epidemic structure and indicate that most CF strains are a random sample of the broader *P. aeruginosa* population. The increased abundance of some CF strains in different geographical regions is a likely product of chance colonisation events followed by adaptation to the CF lung and horizontal transmission among patients.

## Introduction

Studies of the genetic structure of microbial populations are central to understanding the evolution, ecology and epidemiology of infectious diseases [1], [2], [3]. While there are numerous studies describing the genetic structures of pathogen populations, most are based on samples drawn from clinical collections. To great a reliance on clinical samples can bias knowledge of population structure and generate misleading conclusions – particularly regarding the evolutionary origins of disease causing lineages.

*P. aeruginosa* is a metabolically versatile bacterium capable of surviving in aquatic, plant, animal and human-associated habitats [4], [5], [6], [7], [8]. Although common in soil and water [9], [10], several studies suggest a more restricted environmental niche partitioning [11], [12]. Indeed, detecting *P. aeruginosa* in drainage systems and medical equipment within domestic and hospital settings likely reflects a related niche in the natural environment [12], [13], [14].

*P. aeruginosa* causes a wide variety of diseases in animals, including wound infections in all species, otitis externa in cats and dogs, respiratory infections in cats, dogs, birds and ungulates, sepsis in poultry and genital infections in horses [4]. It is also an important opportunistic human pathogen causing skin and soft tissue infections in diabetics, severe surgical site and wound infections for burns patients, ventilator-associated pneumonia in the critically ill and severe sepsis amongst cancer and other immunocompromised patients [15].

Notably, *P. aeruginosa* is the most common bacterial pathogen, and a leading cause of morbidity and mortality in patients with cystic fibrosis (CF) [16]. Most *P. aeruginosa* infections in CF patients are thought to be caused by unique strains acquired from the natural environment. However, recent studies show unrelated CF patients can share strains that are indistinguishable from one another by modern molecular DNA typing techniques [17]. Whilst some of these *P. aeruginosa* strains are associated with patients attending CF clinics within the same or neighbouring regions [18], [19], others, such as the Liverpool epidemic strain (LES) and Clone C, affect patients on different continents [20], [21]. We demonstrated recently that a similar situation exists in Australia [22]. Approximately 60% of CF patients residing in the state of Queensland and infected with *P. aeruginosa* harbour one of three genotypes. Two strains, allocated by multilocus sequence typing (MLST) as sequence types (ST)-649 (AUST-01) and ST-775 (AUST-02), are dispersed widely throughout Australian CF clinics, whereas the third MLST genotypic strain, ST-801 (AUST-06), is limited largely to CF patients in Queensland.

The abundance of some CF strains suggests tropism for the CF airway, person-to-person transmission, and/or frequent exposure to common environmental strains. Recent studies found that host specificity may be important [23], [24], while cross-infection is supported by the reduction in new cases following strict patient segregation [25], [26]. Nevertheless, the origins of these highly abundant CF strains are unclear and could simply reflect their frequency in other settings. For example, Clone C is found in both CF and non-CF patients, and in the natural environment of countries throughout the northern hemisphere [21], [27], [28], [29].

Although much of the recent focus has been upon CF-associated *P. aeruginosa* strains, understanding the genetic structure of *P. aeruginosa* populations from a global perspective is highly desirable. Quantifying the diversity of specific house-keeping genes by MLST analyses is a powerful approach for understanding the evolution of the core genome and the processes that shape strain biodiversity. From a limited number of studies, it appears that *P. aeruginosa* has a non-clonal, epidemic population with a few widely distributed and frequently encountered strains [29], [30], [31], [32]. However, even the most comprehensive studies published to date have included either only a small proportion of isolates from non-hospital environmental settings, or isolates collected across large geographical regions over extensive time periods [29], [32]. Consequently the origin and evolution of CF strains remains uncertain.

We therefore sought to describe the population genetic structure of *P. aeruginosa* employing an extensive regional strain collection from natural environmental habitats, animal clinical samples and both CF and non-CF human hosts. In particular, we examined the relationship between environmental isolates collected from a river system and highly abundant CF strains found in patients from the same region. Using an MLST-based approach we discovered that the genetic diversity encompassed in our regional sample was no different from the global *P. aeruginosa* pool. Indeed, our sample included highly successful dominant genotypes defined in previous studies and isolated from different geographical regions. We observed a highly-recombining population with limited evidence of an association between genotype and ecological setting. Taken together, our data indicate CF strains are a random sample of a diverse *P. aeruginosa* population and suggest that the various CF strains abundant in different geographical regions are best explained by chance colonisation events followed by adaptation to the CF lung and local transmission.

## Materials and Methods

### Ethics Statement

The University of Queensland Animal Ethics Committee and Human Research Ethics Committees at the Royal Brisbane and Woman’s Hospital, Royal Children’s Hospital, The Prince Charles Hospital, Mater Adult and Children’s Hospitals, Brisbane, and the Gold Coast Hospital, Southport, Queensland, Australia each approved the study. Where applicable, all participants (or their guardian) provided written informed consent.

### *P. aeruginosa* Isolate and Collection Strategies

Overall, 501 *P. aeruginosa* isolates cultured from the environment (n = 106), animal clinical specimens (n = 106), and non-CF (n = 129) and CF patients (n = 160) in South East Queensland, Australia were examined (Table S1). Four-hundred and eighty-seven (97%) isolates were collected prospectively between February 2006 and February 2009, and 14 (13 non-CF, 1 animal) were cultured between 2002 and 2005. Their DNA was extracted, they were confirmed as *P. aeruginosa* by real-time polymerase chain reaction (PCR) assay as described previously [33], and then the isolates and their extracted DNA were stored at −80°C until further testing.

**Figure 1:**
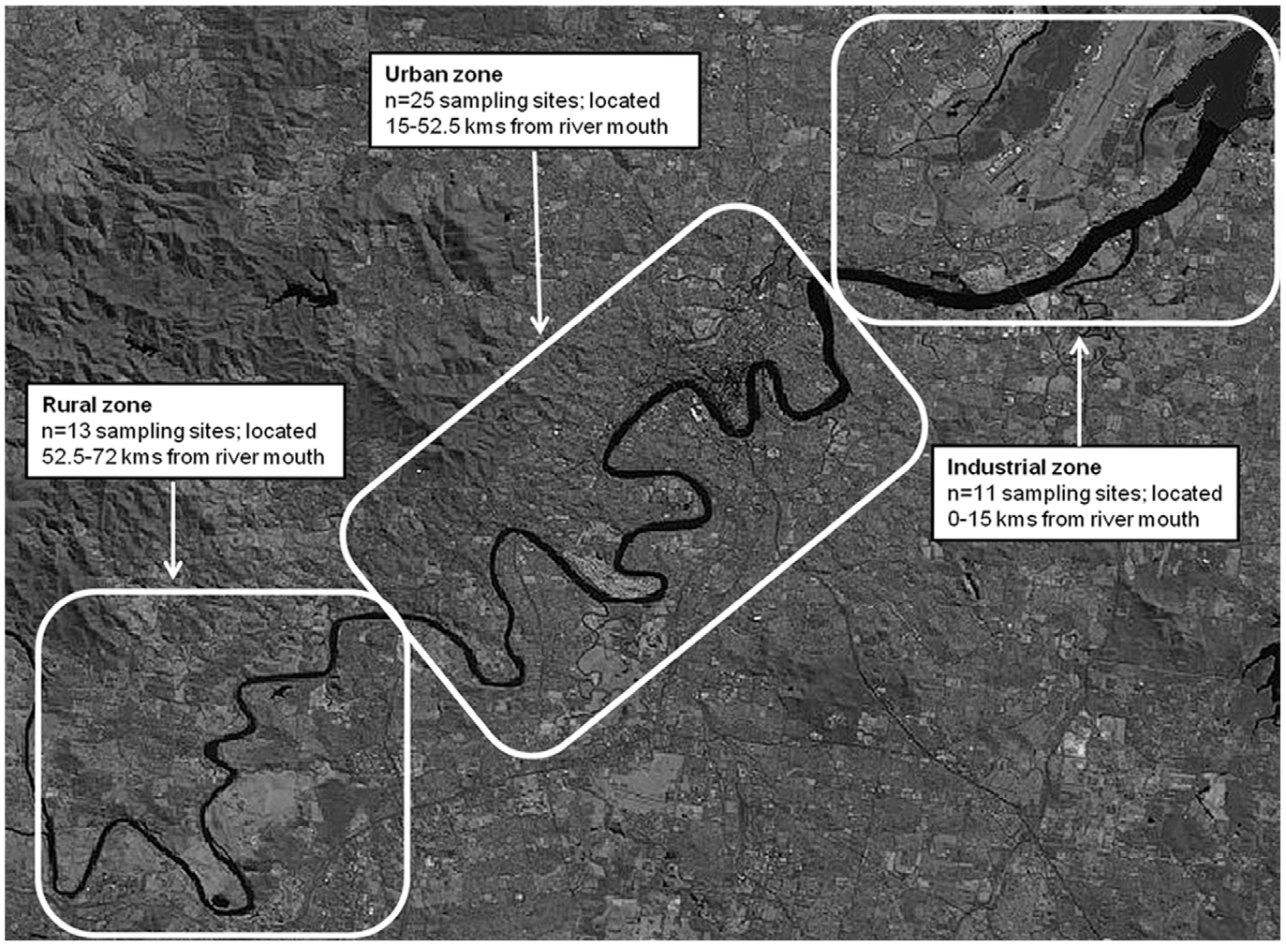
Map (1∶136,268) of the Lower Brisbane River showing land use categories.

Riverine isolates were cultured from surface water grab samples and/or air-water interface swabs from three South East Queensland river systems, the Lower Brisbane, Logan and North Pine Rivers. Lower Brisbane River isolates were sampled from 49 collection sites at 1.5 km intervals extending from the river mouth to 72 kms upstream. This geographical region included industrial, urban, and rural habitats (Figure 1). Logan River isolates were sampled from 14 collection sites over 126 kms, while isolates from the North Pine River comprised 12 collection sites over 52 kms. Logan and North Pine River collection sites extended from the mouth to the headwaters and were associated with predominantly rural and urban habitats (Figures S1 and S2). All riverine samples were categorised in terms of the distance they were collected from the river mouth [i.e. at 5 km intervals], and site land use [i.e. industrial, urban, or rural] (Table S1).

One-hundred ml of each environmental water sample was passed initially through a 47mm, 0.45 micron cellulose filter. Filtered water and swab samples were cultured onto MPAC agar (BD BBL™, North Ryde, Australia) at 42°C for 24 to 48-hours. All suspect colonies and six random subcultures (for heavily contaminated cultures only) were inoculated onto MacConkey Agar No. 2 (Oxoid Australia Pty. Ltd., Adelaide, Australia). Presumptive *P. aeruginosa* isolates were screened by real-time PCR and, once confirmed, were genotyped using enterobacterial repetitive intergenic consensus (ERIC)-PCR typing assays [34].

In all, 33 (67%) of the 49 Lower Brisbane River collection sites grew *P. aeruginosa* resulting in 251 individual colonies [median (range) colonies/positive collection site (PCS) = 4 (1–25)], and 61 different ERIC-PCR genotypes [median genotypes/PCS = 1 (1–6)]. In addition, seven (27%) of 26 Logan and North Pine River sample collection sites were culture positive generating 223 individual colonies [median colonies/PCS = 4 (1–67)] and six genotypes [median genotypes/PCS = 1(1–1)]. One isolate from each ERIC-PCR genotype and all positive collection sites were selected for MLST [Lower Brisbane River (n = 64), Logan River (n = 5) and North Pine River (n = 1)].

Other environmental *P. aeruginosa* isolates used in the study consisted of: (i) 25 isolates cultured from prospectively collected municipal pool (n = 19), tap (n = 5) and tank (n = 1) water samples submitted to the Queensland Health Public Health Microbiology Laboratory for routine monitoring between September 2008 and January 2009; and (ii) 11 household sink isolates collected from nine non-CF households during a separate environmental investigation in 2007. Thus, the 106 environmental isolates submitted for MLST comprised 70 (66%) isolates cultured from three river systems, 25 (24%) from pool, tank and tap water specimens, and 11 (10%) derived from household sinks.

MLST was also performed on 106 *P. aeruginosa* isolates collected from various infection sites involving 10 domestic or native animal species (57 [54%] dogs, 24 [23%] horses, 11 [10%] cats, six possums [5.5%], three snakes [3%], and one each from a bird, cow, goat, koala, and a lizard [n = 106 individual animals]) and submitted to IDEXX Laboratories Pty. Ltd. (East Brisbane) and The University of Queensland Veterinary Diagnostic Laboratory during 2005–2009.

MLST was performed on 129 non-CF human *P. aeruginosa* isolates collected from 129 individual adult and paediatric patients receiving healthcare in eight hospitals in South East Queensland and included a variety of healthcare-associated and community-acquired infections (38 (30%) respiratory, 20 (16%) urine, 19 (15%) superficial wound, 18 (14%) blood, 18 (14%) ear, 14 (11%) other sterile site, and 2 (1.5%) eye). Of the 38 non-CF respiratory isolates, 14 were from patients with bronchiectasis (n = 13) and chronic obstructive pulmonary disease (n = 1), and each were treated at the same hospital, including the same inpatient wards, as those attending the primary regional adult CF clinic.

The 160 CF isolates were collected from 145 sputum samples from 145 patients participating in a national multi-centre cross-sectional prevalence study [22]. All isolates were screened initially using ERIC-PCR genotyping assays. A representative range of abundant local and randomly assigned unique *P. aeruginosa* genotypes underwent MLST analysis. Strains selected for MLST were categorised into three groups depending on their overall prevalence within the Queensland CF patient population: (i) major or common CF strains [ST-649 (AUST-01), ST-775 (AUST-02) and ST-801 (AUST-06)] found in ≥10% of patients (n = 35 isolates); (ii) 21 minor CF strains, including two ST-17 (Clone C) isolates, detected in clusters of 1–5% of patients (n = 50 isolates); and (iii) 75 unique CF strains from individual patients, including a single LES isolate [22].

### Multilocus Sequence Analysis

PCR amplification, bi-directional sequencing, and ST assignment was performed in accordance with the *P. aeruginosa* PubMLST website [27]. Evolutionary parameters, including the number of polymorphic nucleotide sites and amino acids, the ratio of nonsynonymous to synonymous amino acid substitutions (dN/dS ratio), and the G+C content of each locus were calculated using START2 version 0.9.0 beta software and the *P. aeruginosa* PubMLST website [27], [35], [36].

### Methods of Analysis

#### Rarefaction and diversity analyses

Rarefaction, coverage, diversity and richness was estimated for the Lower Brisbane River culture collection, because of its relatively large size and the likelihood that this collection represented an unbiased sample of *P. aeruginosa* isolates from the local natural aquatic environment. Rarefaction analysis was performed using MOTHUR v1.5.0. [37]. Isolates were selected randomly to determine the number of unique STs and operational taxonomic units (OTU; a group of closely related isolates) generated from the isolates drawn. This procedure was repeated 1,000 times and the average estimate was used to generate rarefaction curves.

Simpson’s Index of Diversity (the probability that two randomly selected isolates from a sample belong to the same genotype) [38], and Shannon’s Diversity Index (the uncertainty of predicting the genotype of any randomly selected isolate) [39] were used to compare isolates from different natural environmental niches, locations or land use regions. Analysis of Lower Brisbane River sample richness was performed using the Chao 1 richness estimator [40]. The permutational multivariate analysis of variance (PERMANOVA) test implemented in PRIMER v6.1.12 was used to test for differences in genetic diversity between ecological setting and between sample collection site categories (distance or land use) [41]. Statistical significance, derived from permutation testing, was set at *P*<0.05. Visual comparisons of isolates collected from each ecological source were also performed by multi-dimensional scaling (MDS) using PRIMER software.

#### Recombination

Recombination was detected using the likelihood permutation test with LDhat v2.1 software [42]. LDhat also allowed estimates of population-based recombination frequencies. Analysis was performed on individual loci and concatenated sequences of all seven housekeeping genes, while the crossing-over model analysed bi-allelic sites with a frequency of at least 0.1. The LDhat estimate of ρ ( = 2*Ne*Recombination rate) was divided by θ ( = 2*Ne*Mutation rate) to derive the ratio of recombination to mutation (R/M). The relative contributions of recombination and point mutation were also calculated using the clonal diversification method [43]. Point mutations were characterised as single nucleotide polymorphisms (SNPs) occurring in unique alleles. Polymorphism arising through recombination included all alleles showing multiple nucleotide polymorphisms and alleles with SNPs that occurred in more than one ST outside of the BURST group (BG) of interest [44]. Analysis was performed on BGs showing three or more STs, including all single locus-variants (SLVs) and double-locus variants (DLVs) respectively.

#### eBURST analysis and minimum spanning tree construction

eBURST v3 helped detect BGs and investigate evolutionary relationships between all STs collected from the four ecological settings [44]. A stringent group definition inferred clonal relationships between each ST, whereby STs sharing at least six alleles were allocated to the same BG. The bootstrap method (1,000 resamplings) assessed BGs containing at least three different STs.

PHYLOViZ v1.0 software, which uses goeBURST, a recently refined version of the eBURST algorithm, generated a Minimum Spanning Tree (MST) of the entire population [45]. Evolutionary relationships were explored using up to triple-locus variants (TLVs). The ST node dimensions were plotted relative to the number of isolates within a ST.

#### Phylogenetic reconstruction

Maximum likelihood (ML) trees were constructed from concatenated nucleotide sequences using TREE-PUZZLE v5.2 [46]. The Shimodaira-Hasegawa (SH) test compared tree topologies [47], while ClonalFrame v1.1 reconstructed trees by a Bayesian approach, which identified and removed parts of the sequence alignment subject to recombination. This helped preserve ancestral relationships under a model of diversification by point mutation alone [48].

### Comparisons with the PubMLST Database

STs derived in all four ecological settings during our study were compared with the whole PubMLST database (n = 1070 STs; October 2011) [27]. STs were analysed with a stringent group definition, and an eBURST population snapshot showing all SLV relationships and unrelated singletons was created using a group definition of zero. Statistical (PERMANOVA) and visual (MDS) comparisons of all non-duplicate STs appearing in the current study and PubMLST database were also performed.

## Results

### MLST and Evolutionary Parameters

Overall, 274 unique STs were identified amongst the entire 501 *P. aeruginosa* isolate collection. Strikingly, 190 (69%) were novel STs not previously described in the *P. aeruginosa* PubMLST database [27]. Two STs (one CF and one non-CF isolate) contained a one-base pair insertion/deletion (indel) within the mutL locus (presumed mutator strains) and were excluded, leaving 272 STs for further evolutionary analyses.

There were 342 polymorphic nucleotides within concatenated *P. aeruginosa* housekeeping genes (Table 1). The number of distinct alleles detected at each locus ranged from 35 (nuoD) to 62 (trpE), and the proportion of polymorphic nucleotides ranged from 9.6% (nuoD) to 14.3% (ppsA). Nucleotide polymorphisms resulted in a mean of eight (range: 2–25) amino acid changes per locus, however most were synonymous mutations with correspondingly low dn/ds (mean = 0.0271) ratios.

**Table 1:** Genetic diversity and evolutionary parameters of the 272 *Pseudomonas aeruginosa* sequence types.

MLST locus	Size (bp)	Mean %G+C content	Number of alleles	Number of variable nucleotides (%)	Polymorphic amino acid changes	*dn/ds* ratio^1^
*acsA*	390	68.8	53	41 (10.5)	3	0.0063
*aroE*	498	70.5	52	63 (12.7)	25	0.1237
*guaA*	373	65.8	49	36 (9.7)	4	0.0128
*mutL*	442	67.3	46	59 (13.3)	11	0.0167
*nuoD*	366	63.3	35	35 (9.6)	2	0.0038
*ppsA*	370	66.8	45	53 (14.3)	5	0.0143
*trpE*	443	66.2	62	55 (12.4)	5	0.0122
Mean	411.7	66.9	49.1	48.9 (11.9)	7.9	0.0271

1 Ratio of non-synonymous to synonymous amino acid substitutions.

### The Genetic Structure of Environmental *P. aeruginosa*

For reasons outlined in the Methods, we defined the population structure and diversity of *P. aeruginosa* in the environment by analysing isolates from the Lower Brisbane River. An isolate from each ERIC-PCR genotype and all positive collection sites were genotyped by MLST resulting in 58 STs. A dataset of 251 concatenated housekeeping gene sequences was then constructed assuming that each ERIC-PCR type belonged to only one ST [34].

#### Diversity

**Figure 2:**
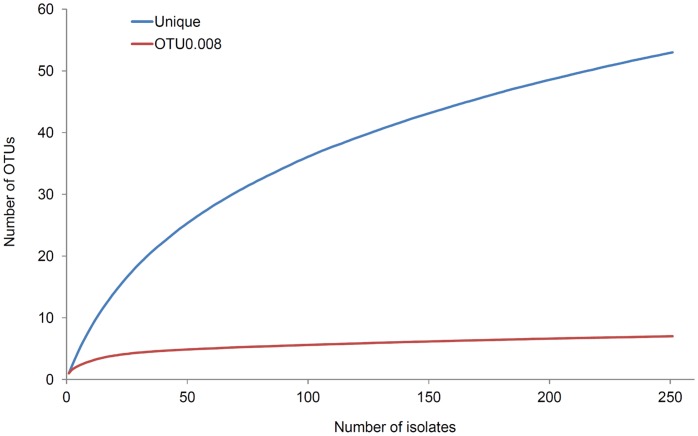
Rarefaction curves showing diversity by unique sequence type (ST) and Operational Taxonomical Unit (OTU). OTUs contained closely related STs, which were separated by the mean pairwise distance of the population, 0.008 (23/2874 nucleotides).

Rarefaction analysis demonstrated the high proportion of unique STs within the Lower Brisbane River sample. Despite the large sample size the rarefaction curve for unique STs was not saturated (Figure 2). We grouped STs into OTUs containing closely-related STs separated by a pairwise distance of 0.008 (23/2874 nucleotides), which was the mean pairwise difference among the population. Figure 2 shows that fewer than 50 randomly selected isolates were required for this rarefaction curve to be saturated, indicating that the population genetic diversity had been covered extensively within our sample of 251 isolates.

Table 2 shows the number, diversity and richness indices, and PERMANOVA results for these 251 isolates originating from different distances and land use categories along the Lower Brisbane River. Thirty (68%) of the 44 culture positive samples contained only one genotype, while only eight STs were isolated from multiple sample collection sites (median = 2, range: 2–4). These observations were supported by high measures of diversity (i.e. low Simpson’s and high Shannon’s indices) for most distance groups and land use categories. Similarly, PERMANOVA analysis revealed no significant difference between the genetic diversity of *P. aeruginosa* isolates from each distance or land use category. An exception was isolates collected from industrial sites, which showed greater levels of sample richness (the ratio of the number of STs to the number of isolates  = 0.53) than isolates detected at urban or rural sites. Importantly, 77% of these industrial isolates were sampled from a single, major sewerage outfall site resulting in six different STs; however, one ST (ST-252) was isolated on 8 occasions. Thus, even though the richness of the industrial isolates was higher, the diversity of the industrial isolates was lower (lower Shannon’s index with confidence intervals that did not overlap with urban or rural isolates).

**Table 2:** Genetic diversity and recombination amongst the 251 *Pseudomonas aeruginosa* isolates Shannon’s Index (95% CI) collected from the Lower Brisbane River.

Category	Grouping^1^	Number of isolates	Number of STs	Number ofOTUs^2^	Simpson’s Index(95% CI)	Shannon’s Index(95% CI)	Chao 1 richnessestimate (95% CI)	PERMANOVAPseudo-F value^3^	R/M^4^
**Distance from river mouth**	<25 km	73	17	5	0.16 (0.11–0.22)	2.2 (1.9–2.4)	44 (26–111)	2.34 (0.076)	1.9
	25–50 km	80	19	4	0.16 (0.11–0.21)	2.2 (2.0–2.5)	106 (45–313)		3.7
	>50 km	98	23	4	0.08 (0.05–0.1)	2.7 (2.6–2.9)	151 (63–431)		3
**Land usage**	Industrial	17	9	4	0.24 (0.04–0.45)	1.7 (1.1–2.2)	16 (9–51)	1.79 (0.132)	4
	Rural	98	23	4	0.08 (0.05–0.1)	2.8 (2.6–2.9)	26 (23–38)		8.5
	Urban	136	27	5	0.10 (0.08–0.12)	2.6 (2.5–2.8)	44 (31–91)		4
**Total**	N/A	251	53	7	0.04 (0.04–0.05)	3.4 (3.3–3.6)	72 (60–109)	N/A	

1 N/A, Not applicable.

2 OTU, operational taxonomic unit containing groups of isolates that varied by a pairwise distance of <0.008.

3 *P-*values are presented in parentheses.

4 R/M, ratio of recombination to mutation; the LDhat likelihood permutation test *P* values were all <0.001.

#### Recombination

High levels of recombination contributed to the marked diversity observed in environmental *P. aeruginosa* isolates from the Lower Brisbane River (Table 2). Population-scaled recombination rates varied between different distances and land use categories, and overall nucleotide substitution was at least 1.9 times more likely to have occurred by recombination than by point mutation.

### Comparison of *P. aeruginosa* Isolates from Different Ecological Settings

We also determined whether the genetic diversity of isolates collected from CF patients was different from that observed in other local ecological settings (i.e. the natural environment, animals and in non-CF patients). Of particular interest were the origins of highly abundant CF strains detected in a recent epidemiological investigation [22]. Population-scaled recombination rates, evolutionary relationships and phylogenetic analyses were also conducted on isolates collected from each of these different settings.

#### Diversity

**Figure 3:**
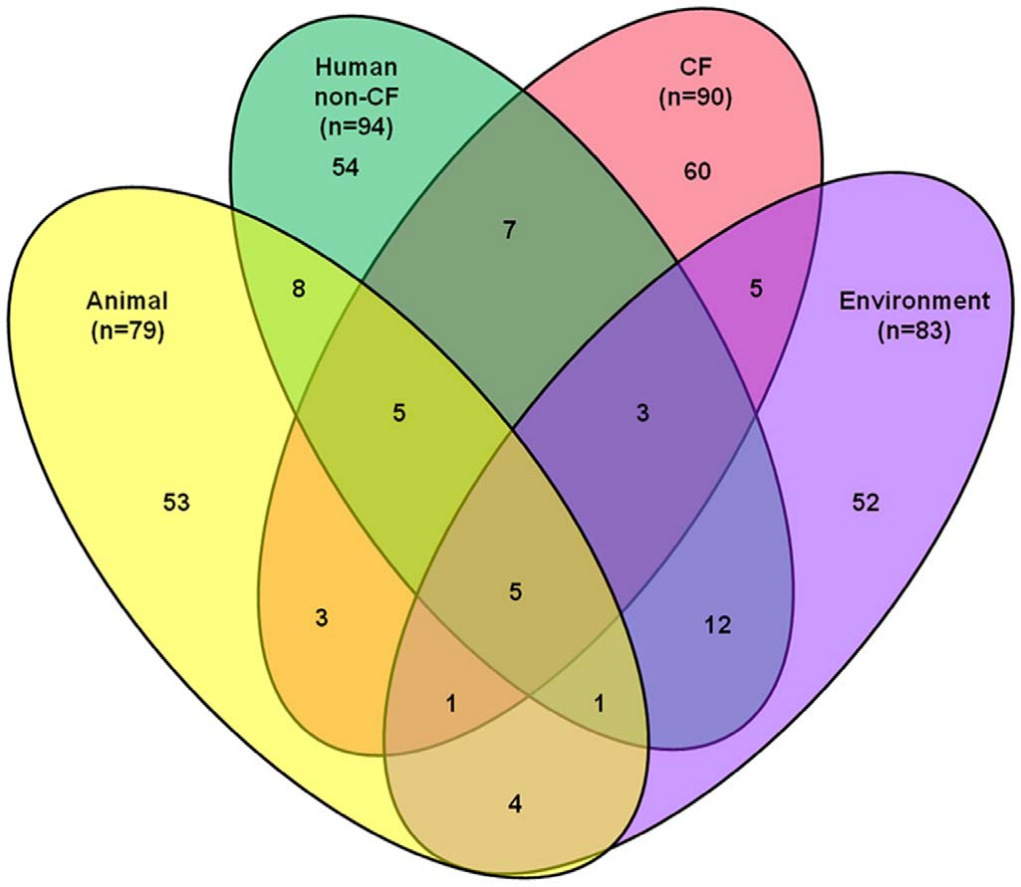
Venn diagram showing the number of individual sequence types (STs) detected in each of the ecological niches. C: STs detected in cystic fibrosis patients; Human non-CF: STs detected in non-cystic fibrosis patients; Animal: STs detected in animals; Environment: STs detected in environmental samples.

The 272 individual *P. aeruginosa* STs were distributed between environmental (n = 83), animal (n = 79), non-CF clinical (n = 94) and CF patient (n = 90) settings. Figure 3 shows that only 53 (19%) STs were shared among different ecological settings. Furthermore, five STs were detected in all four ecological settings (ST-155, ST-179, ST-253 (PA14), ST-266 and ST-381; Table S1), each of which had previously been detected elsewhere in the world [19], [27], [29], [31], [49], [50], [51], [52], [53].

Consistent with the Lower Brisbane River findings we observed no clustering of STs in CF patients compared with STs from broader ecological settings, suggesting that STs isolated from the CF lung are a random subsample of the overall population (Table 3). Initial PERMANOVA analysis of isolates from all ecological settings showed a significant difference in genetic diversity, although the contribution of the ecological setting to the variance was minimal. However, this finding was influenced mostly by the environmental and animal isolates, which each showed significant differences in genetic diversity compared to all other isolates, and a higher number of OTUs and greater degree of diversity. In contrast, we observed no statistically significant differences in ST variation amongst CF and non-CF human isolates when compared to isolates from all other ecological settings. Visual MDS comparisons of isolates from environmental, animal, and human non-CF and CF clinical settings also supported these findings (data not shown).

**Table 3:** Genetic diversity amongst *Pseudomonas aeruginosa* genotypes collected from each ecological setting, all Queensland sources and the *Pseudomonas aeruginosa* PubMLST database.

	Total numberof STs	Number of non-duplicate STs	Number of OTUs^1^	Simpson’s Index (95% CI)^2^	Shannon’s Index (95% CI)^2^	PERMANOVA Pseudo-F value^3^
Environment	83	52	15	0.33 (0.23–0.43)	1.6 (1.3–1.9)	4.87 (0.001)
Animal	79	53	11	0.48 (0.36–0.61)	1.2 (0.9–1.5)	2.16 (0.010)
Non-CF human	94	54	8	0.57 (0.45–0.68)	1.0 (0.7–1.2)	0.77 (0.581)
CF	90	62	5	0.60 (0.48–0.72)	0.8 (0.6–1.0)	1.88 (0.086)
Queensland - total	272	188	12	0.53 (0.45–0.62)	1.1 (0.9–1.3)	
*P. aeruginosa* PubMLST database	1070	798	39	0.41 (0.37–0.45)	1.5 (1.4–1.6)	1.79 (0.094)^4^

1 OTU, operational taxonomic unit containing groups of isolates that varied by a pairwise distance of <0.008.

2 Simpson’s and Shannon’s indices were calculated for OTUs, because only non-duplicate STs were included for analysis.

3 Comparison of each ecological source with the total dataset; *P-*values are presented in parentheses.

4 Comparison of non-duplicate STs between Queensland MLST dataset and global dataset (*P. aeruginosa* PubMLST database [27]).

#### CF and non-CF human genotypes in other ecological settings

Twenty-eight (31%) of 90 CF STs were observed in environmental isolates and/or in animal and non-CF human infections (Table 4). All CF STs identified from other ecological settings represented either unique or minor CF cluster strains. In contrast, the three major or dominant CF strains, AUST-01 (ST-649), AUST-02 (ST-775) and AUST-06 (ST-801) were confined to CF patients.

**Table 4:** Relationships between local cystic fibrosis (CF) *Pseudomonas aeruginosa s*equence types (STs) and identical STs detected in other ecological settings.

P. aeruginosa ST in CF patients^1^	Total STs	CF STs detected in ≥1 niche (%)	Environment (%)	Animal (%)	Non-CF (%)
Unique	64	17 (27)	11 (17)	7 (11)	10 (16)
Minor	23	11 (48)	3 (13)	6 (26)	9 (39)
Major	3	–	–	–	–
Total	90	28 (31)	14 (16)	13 (14)	19 (21)

1 Unique: *P. aeruginosa* strains detected in individual Queensland CF patients; Minor: CF strains detected in clusters of 1–5% of Queensland CF patients; Major: CF strains [ST-649 (AUST-01), ST-775 (AUST-02) and ST-801 (AUST-06)] found in ≥10% of Queensland CF patients [22].

Twenty-two environmental, animal and non-CF human clinical STs were represented by at least three (range 3–16) isolates (Table S1). Of these, 10 were also detected in CF patients infected with either unique (ST-27, ST-147, ST-244, ST-253, ST-266, ST-381, ST-471) or minor (ST-155, ST-179, ST-274) CF strains. Interestingly, 20 (91%) of these STs were additionally isolated from more than one ecological setting. The two exceptions were a predominant equine genital strain, ST-883 [54], and a swimming pool isolate ST-890. Likewise, seven (64%) of the 11 most frequently encountered environmental and animal STs (at least three isolates detected) were also present in non-CF patients (ST-155, ST-179, ST-252, ST-253, ST-313, ST-381, ST-385).

The 14 isolates derived from patients with non-CF chronic lung disease comprised 14 different STs, including three STs identical to unique and minor shared CF strains (ST-253 (PA14), ST-274, ST-807). In addition to ST-253 (PA14), ST-155 and ST-179, other well known STs were also identified in the current study. These included ST-17 (Clone C) from non-CF and CF patients; ST-146 (LES) from a CF patient; ST-27 from household drains, non-CF patients and a CF patient; ST-235 from non-CF patients; and ST-274 from animals, non-CF and CF patients.

#### Recombination

Evidence of recombination was detected in five of seven loci (Table 5), and nucleotide substitution was approximately 19 times more likely to have occurred by recombination than point mutation. The clonal diversification method also confirmed that recombination played an important role in determining short-term genetic diversity. Of 18 variant alleles identified, 14 (78%) arose by recombination and four emerged by point mutation (Table 6). Overall, recombination generated new alleles 3.5 times more frequently than mutation, and the per nucleotide site r/m parameter was 9.5∶1.

**Table 5:** Estimates of population-based mutation (θ) and recombination (ρ) rates among the 272 *Pseudomonas aeruginosa* sequence types using LDhat.^1^

Gene	Nucleotides analysed	Parameters^2^						Likelihood permutation test^3^
		SS	Average PWD	θ	ρ	R/M		
Concatenated	2874	36	14.4	5.82	110		18.9	<0.001
*acsA*	390	8	3.4	1.76	65		37	<0.001
*aroE*	495	10	4.4	2.2	11		5	0.004
*guaA*	372	7	2.1	1.56	35		22.4	0.024
*mutL*	441	4	1.7	0.9	23		25.5	<0.001
*nuoD*	366	7	1.9	1.69	250		148.3	0.648
*ppsA*	369	4	1.4	0.92	70		76.1	0.073
*trpE*	441	11	4.4	2.34	40		17.1	<0.001

1 Only polymorphic sites with exactly two alleles were analysed.

2 SS: number of segregating sites; PWD, pairwise distance; R/M: per nucleotide site recombination to mutation parameter.

3 Likelihood permutation test results for the influence of recombination are presented as *P*-values.

**Table 6:** Relative contributions of recombination and mutation to recent clonal divergence.

Total variant alleles	Total nucleotide changes	Recombinational imports		Point mutations	Per fragment r/m parameter^1^	Per site r/m parameter^2^
		Variant alleles	Total nucleotide differences			
18	43	14^3^	38	4	3.5∶1	9.5∶1

1 Per fragment r/m parameter: Estimated ratio of recombination to mutational events per gene fragment.

2 Per site r/m parameter: Estimated ratio of the recombinational to mutational changes at an individual nucleotide site.

3 Variant alleles, which arose through recombination and consisted of seven alleles with multiple nucleotide polymorphisms, and seven alleles showing single nucleotide polymorphisms.

#### eBURST analysis and minimum spanning tree construction

The 272 STs and related SLVs were grouped by eBURST into five BGs containing three to 11 STs and another 26 BGs of two STs each. In addition there were 196 singleton STs that lacked any SLVs (Table S1, Figure S3). The largest BG had 11 STs (43 isolates) originating from all four ecological settings and included the major CF strain ST-775 (AUST-02), two minor CF strains, three unique CF strains, and the equine genital strain ST-883. Of the other major CF-related strains, ST-649 (AUST-01) was a singleton ST, while ST-801 (AUST-06) shared BG-25 with an animal isolate ST-4. Frequently encountered genotypes ST-155, ST-253 (PA14), ST-266 and ST-274 each belonged to different BGs, while others including ST-179 and ST-381 represented singleton STs.

**Figure 4:**
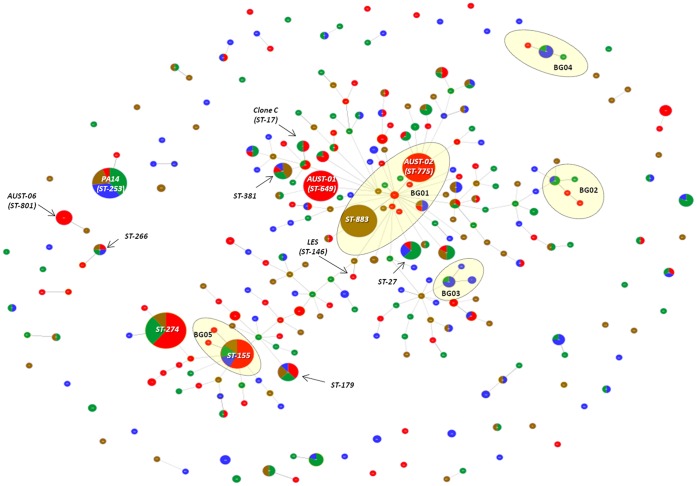
goeBURST Minimal Spanning Tree of the 499 typeable *Pseudomonas aeruginosa* isolates (n = 272 sequence types). All sequence types (STs) were grouped up to the triple-locus variant level. Each circle corresponds to an individual ST and the dimensions of each circle are relative to the number of isolates belonging to that ST. Red, green, brown and blue circles represent isolates collected from patients with cystic fibrosis (CF), non-CF patients, animals and environmental samples, respectively. Whenever isolates of the same ST have a different ecological source, the number of isolates derived from the same source is proportional to the respective colour. Yellow coloured zones (labelled BG01 to BG05) represent the five predominant BURST groups that each consisted of three or more STs.

When a MST linking all TLV relationships was constructed, we observed a network of associations (Figure 4). In all, 177 (65%) STs were grouped into a single cluster, while only 18 remained as ungrouped singleton STs. Interestingly, the predicted founder of the largest cluster (minor CF strain ST-241) was also the putative ancestral founder of BG-01.

#### Phylogenetic analysis and tree congruence

To further elucidate the evolutionary relationships between STs we attempted to reconstruct ML phylogenies from individual housekeeping gene sequences and the concatenated sequences of all seven housekeeping genes. In order to test the validity of phylogenetic reconstruction, trees were reconstructed using 48 STs selected to encompass a broad range of genetic diversity. These STs included: (i) at least one ST from each BG, (ii) each of the abundant inter-niche STs, and (iii) a range of minor and major CF strains. Although the number of isolates utilised was supported by rarefaction analysis (Figure 2), this approach was rejected because the likelihood value of the phylogeny reconstructed from concatenated housekeeping gene sequences was lower than the likelihood values of phylogenies reconstructed from individual housekeeping genes. Similarly, the topology of the concatenated gene phylogeny was significantly different from the topology of individual gene phylogenies (SH test, P<0.05 for all pairwise comparisons).

**Figure 5:**
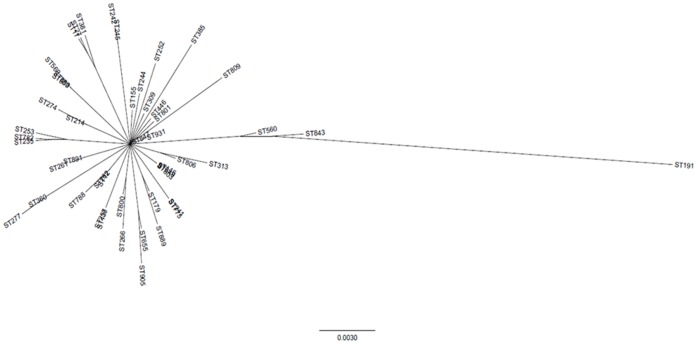
Phylogenetic tree constructed from 48 concatenated guaA, mutL, and nuoD sequences indicating a star-like phylogeny.

In a further attempt to reconstruct a reliable phylogeny that encompassed the core *P. aeruginosa* genome we also compared the phylogeny of individual housekeeping genes to those constructed from all concatenated pairs and triplets of housekeeping genes. A tree reconstructed from concatenated guaA, mutL, and nuoD genes was the only phylogeny whose topology was not significantly different from guaA, mutL, and nuoD individual gene phylogenies. The resulting phylogeny was star-like, indicating evolution from a single common ancestor, or alternatively, a non-informative tree (Figure 5). Overall, there was a lack of congruence between trees constructed from sequences of individual loci indicating that different components of the core genome vary considerably with regards to their ancestry, presumably as a consequence of widespread recombination. Phylogenetic reconstruction using ClonalFrame (which removes portions of the nucleotide alignment subject to recombination) also resulted in a non-informative tree.

### Genetic Diversity and Structure of the Global *P. aeruginosa* Population

Finally, we determined whether the population structure of P. aeruginosa isolates from South East Queensland differed substantially from the population structure observed on other continents. As there were no samples similar to our own that have been analysed by the same MLST genotyping scheme, we compared the genetic diversity of non-duplicate STs from our sample with global non-duplicate STs in the PubMLST database.

**Figure 6:**
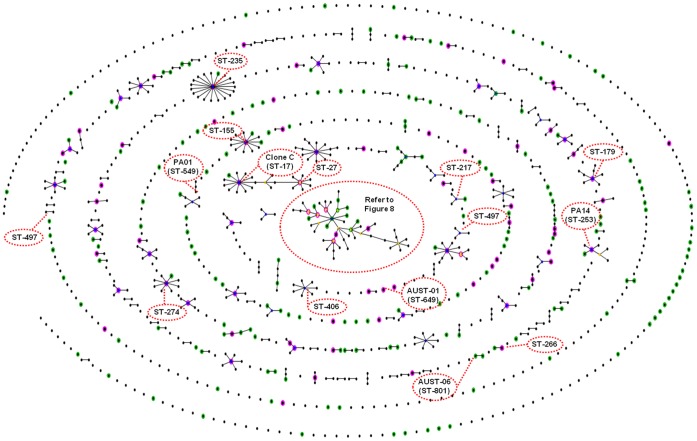
Population snapshot of the 1070 sequence types listed on the *Pseudomonas aeruginosa* PubMLST database (October 2011). Dots represent sequence types (STs), and lines connect single-locus variants. The snapshot shows all BURST groups (connected STs), singleton STs, ancestral founders (blue STs), and subgroup founders (yellow STs). STs with green halos were detected in the current study only; STs showing pink halos were detected in the current study and elsewhere; STs with no halo were not detected in the current study. Line length and singleton ST placement is arbitrary.

**Figure 7:**
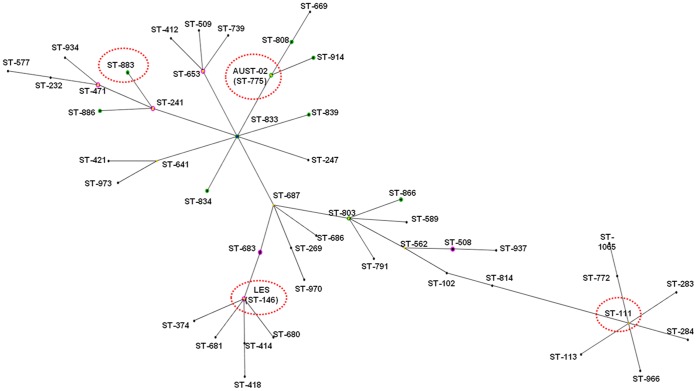
eBURST diagram showing BURST Group 01 when all 1070 sequence types listed on the *Pseudomonas aeruginosa* PubMLST database (October 2011) were analysed. Dots represent sequence types (STs), and lines connect single-locus variants. The diagram shows all ancestral founders (blue STs) and subgroup founders (yellow STs). STs with green halos were detected in the current study only; STs showing pink halos were detected in the current study and elsewhere; STs with no halo were not detected in the current study. Line length is arbitrary.

eBURST analysis of all 1070 STs on the PubMLST database revealed 66 predominant BGs consisting of three or more STs, 79 BGs involving two STs, and 514 singleton STs (Figures 6, 7, and S4). The 272 STs identified in our study were highly representative of the global *P. aeruginosa* population. Our sample contained at least one ST from 76% of the 66 predominant BGs, and 64% of all putative ancestral founding genotypes. The most common lineage detected amongst the 1070 STs was also identical to the largest BG identified amongst the 272 Australian STs. This lineage included a range of important subgroups and strains, such as ST-146 (LES), ST-775 (AUST-02), multidrug resistance associated strain ST-111, and highly abundant equine genital strain ST-883 [54] (Figure 7). The second largest BG was characterised by ST-17 (Clone C) and ST-27. Other important CF strains, such as ST-801 (AUST-06), ST-217 (Manchester strain) and Dutch strains ST-406 and ST-497 occurred within other unrelated BGs, while ST-649 (AUST-01) was again represented by a singleton ST.

Twelve (55%) of the 22 most frequently encountered environmental, animal and non-CF human clinical STs detected in our study (e.g. ST-155, ST-179, ST-253 (PA14) and ST-274) represented founding or sub-founding genotypes for many of the larger BGs, six (27%) belonged to smaller BGs, while only four (18%) were singleton STs.

Statistical comparisons of all non-duplicate STs appearing in the current study and *P. aeruginosa* PubMLST database were also highly supportive of our eBURST-based findings. The degree of diversity within the two datasets was similar, and we observed no significant difference between the genetic diversity in our South East Queensland dataset and global *P. aeruginosa* (Table 3).

## Discussion

This analysis of *P. aeruginosa* genetic diversity, population structure and evolutionary divergence is based upon the most extensive isolate collection obtained so far from a single geographic region. It is also notable for having more than two-thirds of the isolates originating from non-CF settings. The most significant and generally relevant findings include discovery of: (i) an overall lack of association between genotype and ecological setting; (ii) frequent recombination among housekeeping genes; (iii) no difference in diversity at local versus global scales; and (iv) an over-representation of certain STs which included STs identified in previous studies. Overall, the Queensland population of *P. aeruginosa* has a non-clonal, epidemic structure punctuated by a small number of abundant genotypes and large BGs of three or more STs (i.e. clonal complexes). This population structure has been described previously [29], [30], [31], [32]. However, including a substantive set of environmental samples strengthens these observations and provides additional general insights into the population structure and the origin of CF strains.

Consistent with earlier reports, the lack of statistical association between genotype and ecological setting suggests CF strains are a random sample of the overall *P. aeruginosa* population and that potentially all environmental strains can cause infection of the CF lung [21], [29], [31], [32]. In addition, approximately 30% of STs from CF patients were also isolated from other ecological settings. Taken together, these observations emphasise that strictly enforced current infection control strategies within CF clinics and communities will not prevent the acquisition of *P. aeruginosa* strains from the environment [32].

### Recombination

Earlier reports using qualitative methods, including a lack of congruence between phylogenies based on different loci, mosaic genetic structuring, and measures of linkage disequilibrium gave indirect evidence of recombination [29], [30], [32], [55], [56]. More recently, a study involving predominantly clinical isolates from five Mediterranean countries provided evidence of recombination occurring within *P. aeruginosa* populations and that recombination was more a common source of nucleotide substitution than point mutation [31]. Our data similarly demonstrate that alleles are at least 3.5 times more likely to change by recombination than mutation. Moreover, we found significant evidence of recombination among a subset of environmental isolates collected from the Brisbane River. Together, our data indicate that *P. aeruginosa* has a similar structure to other pathogens with highly recombinant populations, such as Neisseria meningitidis, Streptococcus pneumoniae, and Vibrio parahaemolyticus [43], [57], [58].

Not unexpectedly, we found recombination to be sufficiently frequent to destroy any overall phylogenetic information [59]. There was no congruence between trees constructed from sequences of individual loci, indicating that different components of the core genome vary considerably in their ancestry. Consequently, traditional phylogenetic clustering techniques are unsuitable for determining long-term epidemiological relationships and evolutionary pathways for P. aeruginosa. Indeed, employing such approaches, particularly where phenotypic characters are mapped onto gene trees [32], may generate erroneous assumptions [59]. Instead, clustering techniques that associate STs by allelic designation should be used for identifying recent evolutionary patterns.

Recombination is the most likely mechanism for failing to identify an overall association between genotype and ecological setting. The absence of population substructures, such as biovars or delineated ecotypes, suggests that irrespective of strain origin *P. aeruginosa* is evolving as a single cohesive genetic group. A recent study of diversity across the genus Pseudomonas concluded that of all the species, only *P. aeruginosa* conformed to a ‘clear, compact, defined species’ [60]. However, just what maintains this integrity, aside from recombination is uncertain.

### Genotypic Diversity

The discovery of both major and minor CF-related *P. aeruginosa* strains is also consistent with previous reports [19], [20], [21]. However, while previously known STs, such as ST-155, ST-179 and ST-274, were encountered on multiple occasions and in different ecological settings, other abundant STs were uniquely Australian [e.g. ST-775 (AUST-02) and ST-801 (AUST-06)]. This reinforces the hypothesis of CF-related strains being a random subset of the total *P. aeruginosa* population and where chance colonisation events specific to different geographical regions also play a role. Once genetic adaptation to the CF lung occurs [61], [62], a small number of genotypes may develop an enhanced ability for person-to-person transmission. Indeed, evidence of transmission and evolution was also evident in the present study. For example, ST-775 (AUST-02) was isolated on numerous occasions from CF patients and it showed close evolutionary relationships to several STs that are likely to have evolved from this common CF genotype.

The major Queensland CF strains [ST-649 (AUST-01), ST-775 (AUST-02) and ST-801 (AUST-06)] were isolated from CF patients, but not from other sources, suggesting patient-to-patient transmission has occurred. The same holds true for the major equine-associated strain (ST-883) that was isolated exclusively from the horse reproductive tract [54]. These findings are consistent with earlier reports of ST-649 (AUST-01), where despite it being abundant in CF patients, repeated microbiological surveys failed to identify it in the healthcare environment [63]. Such observations are in marked contrast with other previously known CF-associated STs, such as ST-155, ST-179, ST-253 (PA14) and ST-274, that we isolated from many CF patients and multiple other ecological settings.

While isolates belonging to ST-155, ST-179, ST-253 (PA14) and ST-274 are likely to show substantive variation at other loci, particularly among their accessory genes [29], [31], [34], finding STs in Queensland showing identity over approximately 3,000 nucleotides to previously recorded STs is remarkable. While substantiating the existence of highly abundant STs, this study shows that such STs are truly globally distributed and in some cases would appear to have persisted for many years. However, the existence of global genotypes within a freely recombining bacterial population is difficult to reconcile [64]. In the face of recombination, strains or large BGs (clonal complexes) should be transient and yet ST-253 (PA14), for example, was first isolated from a human infection in the United States more than 15-years ago [65]. ST-253 (PA14) is common throughout the Northern Hemisphere [27], [29], [31], [50], and in the present study was isolated on 15 occasions from a broad range of environmental, animal and clinical settings. The most parsimonious explanation invokes an extraordinary population expansion of a small number of lineages in a brief period of time with these abundant genotypes then achieving global representation. However, just what would cause such an explosive dispersal across environments that include river systems in Queensland is far from clear.

The finding of abundant environmental STs has been suggested previously as the cause of their prevalence in CF patients [32], [66]. Our findings both support and question this claim. Indeed, strains such as ST-253 (PA14) are widespread in other ecological settings, thus increasing the chance that they will also infect the CF lung. However, despite their widespread presence in other ecological settings, these abundant strains still represent only unique or minor CF strains present in relatively small clusters of CF patients. In contrast, the most common CF strains in this geographic region [ST-649 (AUST-01), ST-775 (AUST-02) and ST-801 (AUST-06)] were not encountered in other ecological settings. These findings are consistent with a recent Dutch study, which suggested that common CF strains, ST-406 and ST-497, exhibited high-level host specificity for the CF lung microenvironment [24].

Our study offers further insights, when placed in context of the entire *P. aeruginosa* population reflected by the PubMLST database. First, the diversity shown in the present study did not differ significantly from that of the global collection. This is consistent with findings from a Belgian river system [7]. Interestingly though, we did not find a positive relationship between river pollution and the detection of *P. aeruginosa* as reported in the Belgian study [7]. Moreover, approximately 15% of river STs were present at multiple collection sites, including ST-863, which was cultured from both the Lower Brisbane and Logan Rivers. Finding that local diversity was no different than global diversity is important for understanding the apportionment of *P. aeruginosa* diversity across space. Second, although there was no close association between genotype and environment, some STs, many associated with CF, have diverged from a set of closely related STs. While extensive recombination means the overall relationship amongst these large BGs (clonal complexes) is unknown, the existence of such complexes suggests that certain *P. aeruginosa* genotypes shared between CF patients have become specifically adapted to the CF lung [67].

### Strengths and Limitations

The strengths of this study include its temporal and spatial design, the analytical approaches taken, and the extent of our sample collection. Isolates were obtained from a single geographical region over a short timeframe to reduce the confounding effect of population diversification over time and geographical location. Genotypic and evolutionary analyses were undertaken using techniques capable of defining genetic relatedness in a highly recombinant species collected from a diverse range of ecological settings. Meanwhile, the richness of our sample including isolates from diverse non-human origins provided an extensive and unbiased view of population diversity.

There are however some limitations. Firstly, the inhibitory effects of the selective agents present in the MPAC agar may have influenced the recovery of *P. aeruginosa* from the environmental samples. This raises the possibility that some environmental genotypes were excluded from the genotypic diversity and population analyses. However, many of the environmental samples contained high levels of background contamination, meaning that using selective culture medium to improve *P. aeruginosa* detection was unavoidable. MPAC agar shows adequate selectivity, sensitivity and specificity, and is currently recommended by the Joint Standards Australia/Standards New Zealand Committee for the primary isolation and enumeration of *P. aeruginosa* from water samples [68], [69], [70]. Second, the current MLST-based study provides no information regarding broader genomic diversity. Published annotated genome sequences demonstrate that most strains share a conserved core, but have a highly variable accessory genome [71], [72], [73], [74], [75]. The emergence of abundant CF *P. aeruginosa* strains, although poorly understood, may involve subtle core and accessory genomic transitions, but also, prophage and genomic insertions that enhance the potential for niche specialisation, increased competitiveness and enhanced transmission [62], [72], [73], [75]. Future studies aimed at exploring core and accessory genomic elements, protein expression and metabolic fitness in response to different environmental cues, and rates of hypermutation are now planned for this culture collection.

In conclusion, this study represents the most extensive analysis of biodiversity, population structure and evolution of *P. aeruginosa* sampled from multiple ecological niches within a discrete geographical region. We found minimal correlation between genotype and habitat. Furthermore, our sample was representative of global population diversity and contained several highly successful genotypes, which were common to multiple sources and locations internationally. Abundant CF-related strains were however not found in other settings, suggesting host specificity and indirectly supporting widespread cross-infection and/or niche specialisation.

## Supporting Information

Figure S1 **Map (1∶272,536) of the Logan River showing land use categories.**
(TIF)

Figure S2 **Map (1∶136,268) of the North Pine River showing land use categories.**
(TIF)

Figure S3 **Results of eBURST analysis for the 499** ***Pseudomonas aeruginosa*** **isolates.** Total no. of isolates  = 499; Total no. of ST  = 272; No. of loci per isolate  = 7; No. of identical loci for group definition  = 6; Total no. of BURST groups detected  = 31; No. of re-samplings for bootstrapping  = 1000.
(PDF)

Figure S4 **Results of eBURST analysis for the 1070** ***Pseudomonas aeruginosa*** **sequence types listed in the** ***Pseudomonas aeruginosa*** **PubMLST database.** *P. aeruginosa* MLST database: http://pubmlst.org/paeruginosa/; Accessed 04 October 2011; Total no. of STs  = 1070; No. of loci per isolate  = 7; No. of identical loci for BURST group definition  = 6; Total no. of BURST groups detected  = 145; No. of re-samplings for bootstrapping  = 1000. Green coloured STs were detected in the current study only; pink coloured STs were detected in the current study and elsewhere; black coloured STs were detected in the current study.
(PDF)

Table S1 **List of the sources, sampling details, relevant epidemiological, clinical and geographical characteristics, multilocus sequence typing data and results of eBURST analyses for the 501** ***Pseudomonas aeruginosa*** **isolates.**
(XLSX)
