# Supplementary material for: Pseudomonas aeruginosa Exhibits Frequent Recombination, but Only a Limited Association between Genotype and Ecological Setting
Source: PLoS One. 2012 Sep 6;7(9):e44199. doi: 10.1371/journal.pone.0044199 (PMC3435406; doi:10.1371/journal.pone.0044199)
Supplement: Figure S3 — Results of eBURST analysis for the 499 Pseudomonas aeruginosa isolates. Total no. of isolates = 499; Total no. of ST = 272; No. of loci per isolate = 7; No. of identical loci for group definition = 6; Total no. of BURST groups detected = 31; No. of re-samplings for bootstrapping = 1000. (PDF) [file pone.0044199.s003.pdf]

Figure S3. Results of eBURST analysis for the 499 *Pseudomonas aeruginosa* isolates.

Total no. of isolates = 499 | Total no. of STs = 272
No. of loci per isolate = 7 | No. of identical loci for group definition = 6
Total no. of BURST groups detected = 31
No. of re-samplings for bootstrapping = 1000

BURST Group 01: No. of isolates = 43 | No. of STs = 11 | Predicted Founder = ST-241

**ST**	**FREQ**	**SLV**	**DLV**	**TLV**	**SAT**	**Average Distance**	**ST Bootstrap Group**	**Subgroup**
241	2	6	4	0	0	1.4	68%	78%
833	1	5	5	0	0	1.5	37%	47%
775	14	5	5	0	0	1.5	37%	41%
653	1	3	7	0	0	1.7	0%	0%
886	1	2	5	3	0	2.1	0%	0%
834	1	2	4	4	0	2.2	0%	0%
808	1	2	3	5	0	2.3	0%	0%
914	1	2	3	5	0	2.3	0%	0%
883	16	1	6	3	0	2.2	0%	0%
839	1	1	5	4	0	2.3	0%	0%
471	4	1	5	4	0	2.3	0%	0%

BURST Group 02: No. of isolates = 6 | No. of STs = 4 | Predicted Founder = Multiple

**ST**	**FREQ**	**SLV**	**DLV**	**TLV**	**SAT**	**Average Distance**	**ST Bootstrap Group**	**Subgroup**
360	3	2	1	0	0	1.33	27%	0%
861	1	2	1	0	0	1.33	26%	0%
864	1	1	1	1	0	2	0%	0%
862	1	1	1	1	0	2	0%	0%

BURST Group 03: No. of isolates = 7 | No. of STs = 3 | Predicted Founder = ST-252

**ST**	**FREQ**	**SLV**	**DLV**	**TLV**	**SAT**	**Average Distance**	**ST Bootstrap Group**	**Subgroup**
252	4	2	0	0	0	1	29%	0%
924	1	1	1	0	0	1.5	0%	0%
9	2	1	1	0	0	1.5	0%	0%

BURST Group 04: No. of isolates = 7 | No. of STs = 3 | Predicted Founder = ST-385

**ST**	**FREQ**	**SLV**	**DLV**	**TLV**	**SAT**	**Average Distance**	**ST Bootstrap Group**	**Subgroup**
385	5	2	0	0	0	1	31%	0%
443	1	1	1	0	0	1.5	0%	0%
920	1	1	1	0	0	1.5	0%	0%

BURST Group 05: No. of isolates = 16 | No. of STs = 3 | Predicted Founder = ST-155

**ST**	**FREQ**	**SLV**	**DLV**	**TLV**	**SAT**	**Average Distance**	**ST Bootstrap Group**	**Subgroup**
155	14	2	0	0	0	1	35%	0%
786	1	1	1	0	0	1.5	0%	0%
13	1	1	1	0	0	1.5	0%	0%

BURST Group 06: No. of isolates = 5 | No. of STs = 2 | Predicted Founder = None

ST	FREQ	SLV	DLV	TLV	SAT	Distance
266	4	1	0	0	0	1
873	1	1	0	0	0	1

BURST Group 07: No. of isolates = 5 | No. of STs = 2 | Predicted Founder = None

ST	FREQ	SLV	DLV	TLV	SAT	Distance
261	2	1	0	0	0	1
810	3	1	0	0	0	1

BURST Group 08: No. of isolates = 2 | No. of STs = 2 | Predicted Founder = None

ST	FREQ	SLV	DLV	TLV	SAT	Distance
859	1	1	0	0	0	1
858	1	1	0	0	0	1

BURST Group 09: No. of isolates = 3 | No. of STs = 2 | Predicted Founder = None

ST	FREQ	SLV	DLV	TLV	SAT	Distance
655	2	1	0	0	0	1
709	1	1	0	0	0	1

BURST Group 10: No. of isolates = 3 | No. of STs = 2 | Predicted Founder = None

ST	FREQ	SLV	DLV	TLV	SAT	Distance
257	2	1	0	0	0	1
780	1	1	0	0	0	1

BURST Group 11: No. of isolates = 16 | No. of STs = 2 | Predicted Founder = None

ST	FREQ	SLV	DLV	TLV	SAT	Distance
253	15	1	0	0	0	1
828	1	1	0	0	0	1

BURST Group 12: No. of isolates = 2 | No. of STs = 2 | Predicted Founder = None

ST	FREQ	SLV	DLV	TLV	SAT	Distance
848	1	1	0	0	0	1
569	1	1	0	0	0	1

BURST Group 13: No. of isolates = 2 | No. of STs = 2 | Predicted Founder = None

ST	FREQ	SLV	DLV	TLV	SAT	Distance
847	1	1	0	0	0	1
896	1	1	0	0	0	1

BURST Group 14: No. of isolates = 7 | No. of STs = 2 | Predicted Founder = None

ST	FREQ	SLV	DLV	TLV	SAT	Distance
845	3	1	0	0	0	1
17	4	1	0	0	0	1

BURST Group 15: No. of isolates = 2 | No. of STs = 2 | Predicted Founder = None

ST	FREQ	SLV	DLV	TLV	SAT	Distance
843	1	1	0	0	0	1
3	1	1	0	0	0	1

BURST Group 16: No. of isolates = 6 | No. of STs = 2 | Predicted Founder = None

ST	FREQ	SLV	DLV	TLV	SAT	Distance
446	5	1	0	0	0	1
298	1	1	0	0	0	1

BURST Group 17: No. of solates = 3 | No. of STs = 2 | Predicted Founder = None

ST	FREQ	SLV	DLV	TLV	SAT	Distance
245	2	1	0	0	0	1
142	1	1	0	0	0	1

BURST Group 18: No. of isolates = 2 | No. of STs = 2 | Predicted Founder = None

ST	FREQ	SLV	DLV	TLV	SAT	Distance
835	1	1	0	0	0	1
891	1	1	0	0	0	1

BURST Group 19: No. of isolates = 3 | No. of STs = 2 | Predicted Founder = None

ST	FREQ	SLV	DLV	TLV	SAT	Distance
235	2	1	0	0	0	1
141	1	1	0	0	0	1

BURST Group 20: No. of isolates = 3 | No. of STs = 2 | Predicted Founder = None

ST	FREQ	SLV	DLV	TLV	SAT	Distance
223	1	1	0	0	0	1
309	2	1	0	0	0	1

BURST Group 21: No. of isolates = 2 | No. of STs = 2 | Predicted Founder = None

ST	FREQ	SLV	DLV	TLV	SAT	Distance
214	1	1	0	0	0	1
917	1	1	0	0	0	1

BURST Group 22: No. of isolates = 2 | No. of STs = 2 | Predicted Founder = None

ST	FREQ	SLV	DLV	TLV	SAT	Distance
806	1	1	0	0	0	1
805	1	1	0	0	0	1

BURST Group 23: No. of isolates = 5 | No. of STs = 2 | Predicted Founder = None

ST	FREQ	SLV	DLV	TLV	SAT	Distance
174	1	1	0	0	0	1
313	4	1	0	0	0	1

BURST Group 24: No. of isolates = 4 | No. of STs = 2 | Predicted Founder = None

ST	FREQ	SLV	DLV	TLV	SAT	Distance
803	3	1	0	0	0	1
866	1	1	0	0	0	1

BURST Group 25: No. of isolates = 7 | No. of STs = 2 | Predicted Founder = None

ST	FREQ	SLV	DLV	TLV	SAT	Distance
801	6	1	0	0	0	1
4	1	1	0	0	0	1

BURST Group 26: No. of isolates = 19 | No. of STs = 2 | Predicted Founder = None

ST	FREQ	SLV	DLV	TLV	SAT	Distance
209	1	1	0	0	0	1
274	18	1	0	0	0	1

BURST Group 27: No. of isolates = 5 | No. of STs = 2 | Predicted Founder = None

ST	FREQ	SLV	DLV	TLV	SAT	Distance
560	4	1	0	0	0	1
137	1	1	0	0	0	1

BURST Group 28: No. of isolates = 2 | No. of STs = 2 | Predicted Founder = None

ST	FREQ	SLV	DLV	TLV	SAT	Distance
146	1	1	0	0	0	1
683	1	1	0	0	0	1

BURST Group 29: No. of isolates = 3 | No. of STs = 2 | Predicted Founder = None

ST	FREQ	SLV	DLV	TLV	SAT	Distance
931	2	1	0	0	0	1
881	1	1	0	0	0	1

BURST Group 30: No. of isolates = 3 | No. of STs = 2 | Predicted Founder = None

ST	FREQ	SLV	DLV	TLV	SAT	Distance
139	2	1	0	0	0	1
115	1	1	0	0	0	1

BURST Group 31: No. of isolates = 2 | No. of STs = 2 | Predicted Founder = None

ST	FREQ	SLV	DLV	TLV	SAT	Distance
889	1	1	0	0	0	1
884	1	1	0	0	0	1

Singletons: No. of isolates = 304 | No. of STs = 196

2	199	389	798	870	916
5	200	395	800	871	918
6	205	399	802	872	919
7	207	451	807	874	922
8	211	463	809	875	923
10	212	494	821	876	925
11	213	499	822	877	926
12	215	508	825	878	927
14	216	514	826	879	928
15	218	532	827	880	930
16	221	553	829	882	932
27	222	554	830	885	
41	224	555	831	887	
110	225	557	832	888	
114	231	564	836	890	
116	233	571	837	892	
117	234	609	838	893	
128	242	611	840	894	
132	244	612	841	895	
136	259	620	842	897	
143	260	633	844	898	
144	262	645	846	899	
145	270	647	849	900	
147	273	649	850	901	
161	275	685	851	902	
164	277	701	852	903	
166	282	708	853	904	
167	296	776	854	905	
168	299	777	855	906	
169	303	778	856	907	
170	308	779	857	908	
172	316	782	860	909	
173	347	788	863	910	
179	348	789	865	911	
189	377	794	867	912	
191	381	796	868	913	
195	386	797	869	915	
