# Supplementary material for: Pseudomonas aeruginosa Exhibits Frequent Recombination, but Only a Limited Association between Genotype and Ecological Setting
Source: PLoS One. 2012 Sep 6;7(9):e44199. doi: 10.1371/journal.pone.0044199 (PMC3435406; doi:10.1371/journal.pone.0044199)
Supplement: Figure S4 — Results of eBURST analysis for the 1070 Pseudomonas aeruginosa sequence types listed in the Pseudomonas aeruginosa PubMLST database. P. aeruginosa MLST database: http://pubmlst.org/paeruginosa/; Accessed 04 October 2011; Total no. of STs = 1070; No. of loci per isolate = 7; No. of identical loci for BURST group definition = 6; Total no. of BURST groups detected = 145; No. of re-samplings for bootstrapping = 1000. Green coloured STs were detected in the current study only; pink coloured STs were detected in the current study and elsewhere; black coloured STs were detected in the current study. (PDF) [file pone.0044199.s004.pdf]

Figure S4. Results of eBURST analysis for the 1070 *Pseudomonas aeruginosa* sequence types listed on the *Pseudomonas aeruginosa* PubMLST database.

P. aeruginosa MLST database: http://pubmlst.org/paeruginosa/ ; Accessed 04 October 2011

Total no. of STs = 1070
No. of loci per isolate = 7 | No. of identical loci for BURST Group definition = 6
Total no. of BURST Groups detected = 145
No. of re-samplings for bootstrapping = 1000
Green coloured STs were detected in the current study only; pink coloured STs were detected in the current study and elsewhere; black coloured STs were not detected in the current study.

BURST Group 01: No. of STs = 49 | Predicted Founder = ST-833

					**Average**	**ST Bootstrap**	
**ST**	**SLV**	**DLV**	**TLV**	**SAT**	**Distance**	**Group**	**Subgroup**
833	8	15	10	15	2.75	66%	84%
241	7	12	15	14	2.83	24%	68%
653	7	11	11	19	3.02	21%	70%
775	6	13	10	19	3.02	7%	35%
111	6	2	5	35	3.79	16%	87%
687	5	14	17	12	2.81	5%	51%
803	5	10	18	15	2.97	9%	60%
146	5	5	6	32	3.72	6%	69%
247	4	14	11	19	3.08	0%	0%
683	4	8	17	19	3.12	0%	20%
562	3	10	14	21	3.16	0%	18%
641	3	9	13	23	3.33	0%	2%
471	3	8	17	20	3.2	0%	6%
808	3	5	14	26	3.62	0%	3%
834	2	9	18	19	3.41	0%	0%
269	2	9	13	24	3.47	0%	0%
686	2	7	19	20	3.25	0%	0%
680	2	7	7	32	3.81	0%	0%
886	2	6	16	24	3.5	0%	0%
914	2	6	14	26	3.62	0%	0%
772	2	6	6	34	3.85	0%	0%
814	2	6	6	34	3.87	0%	0%
102	2	5	13	28	3.64	0%	0%
412	2	5	12	29	3.89	0%	0%
509	2	5	12	29	3.89	0%	0%
589	2	4	14	28	3.66	0%	0%
866	2	4	14	28	3.66	0%	0%
414	2	4	4	38	4.58	0%	0%
232	2	2	16	28	3.58	0%	0%
508	2	2	9	35	4.04	0%	0%
739	1	9	21	17	3.2	0%	0%
839	1	8	14	25	3.64	0%	0%
934	1	7	16	24	3.45	0%	0%
883	1	7	11	29	3.72	0%	0%
970	1	6	11	30	3.66	0%	0%

BURST Group 01 (continued): No. of STs = 49 | Predicted Founder = ST-833

ST	SLV	DLV	TLV	SAT	Average Distance	ST Bootstrap Group	Subgroup
791	1	5	13	29	3.6	0%	0%
283	1	5	7	35	3.93	0%	0%
284	1	5	5	37	4.04	0%	0%
681	1	5	4	38	4.35	0%	0%
966	1	5	2	40	4.66	0%	0%
113	1	5	2	40	4.68	0%	0%
374	1	4	7	36	4.06	0%	0%
973	1	3	10	34	4.02	0%	0%
421	1	2	11	34	4.2	0%	0%
669	1	2	8	37	4.06	0%	0%
418	1	2	3	42	5.18	0%	0%
1065	1	1	6	40	4.54	0%	0%
937	1	1	5	41	4.33	0%	0%
577	1	1	3	43	4.27	0%	0%

BURST Group 02: No. of STs = 23 | Predicted Founder = ST-17

ST	SLV	DLV	TLV	SAT	Average Distance	ST Bootstrap Group	Subgroup
17	11	2	2	7	2.27	93%	99%
27	7	2	3	10	2.86	31%	91%
187	5	10	4	3	2.22	8%	18%
321	5	7	10	0	2.22	11%	29%
845	4	11	4	3	2.27	4%	19%
339	4	9	2	7	2.59	0%	4%
157	4	9	2	7	2.59	0%	4%
445	4	9	2	7	2.59	0%	4%
449	3	9	7	3	2.45	0%	8%
688	3	8	2	9	3.13	0%	0%
958	3	8	2	9	3.13	0%	0%
202	3	8	2	9	3.13	0%	0%
318	2	11	2	7	2.68	0%	0%
322	2	9	4	7	2.77	0%	0%
636	2	9	4	7	2.77	0%	0%
380	2	8	9	3	2.59	0%	0%
182	2	5	12	3	2.72	0%	0%
334	2	5	4	11	3.22	0%	0%
700	2	5	4	11	3.22	0%	0%
129	2	5	3	12	3.27	0%	0%
119	2	5	3	12	3.27	0%	0%
294	1	8	3	10	3.13	0%	0%
120	1	6	2	13	3.81	0%	0%

BURST Group 03: No. of STs = 20 | Predicted Founder = ST-235

ST	SLV	DLV	TLV	SAT	Average Distance	ST Bootstrap Group	Subgroup
235	19	0	0	0	1	100%	100%
651	5	14	0	0	1.73	2%	18%
989	5	14	0	0	1.73	2%	19%
976	5	14	0	0	1.73	3%	19%
533	5	14	0	0	1.73	7%	18%
1015	5	14	0	0	1.73	11%	18%
696	3	16	0	0	1.84	0%	0%
141	3	16	0	0	1.84	1%	0%
745	3	16	0	0	1.84	1%	0%
323	3	16	0	0	1.84	0%	0%
304	3	16	0	0	1.84	2%	0%
622	3	16	0	0	1.84	0%	0%
457	3	16	0	0	1.84	1%	0%
593	3	16	0	0	1.84	2%	0%
230	3	16	0	0	1.84	1%	0%
534	3	16	0	0	1.84	3%	0%
824	3	16	0	0	1.84	2%	0%
342	3	16	0	0	1.84	3%	0%
660	2	17	0	0	1.89	0%	0%
227	2	17	0	0	1.89	0%	0%

BURST Group 04: No. of STs = 14 | Predicted Founder = ST-244

ST	SLV	DLV	TLV	SAT	Average Distance	ST Bootstrap Group	Subgroup
244	10	3	0	0	1.23	99%	100%
441	5	7	1	0	1.69	20%	19%
766	5	7	1	0	1.69	19%	19%
595	4	8	1	0	1.76	1%	1%
594	4	8	1	0	1.76	2%	1%
336	2	9	2	0	2	0%	0%
990	2	9	2	0	2	0%	0%
630	2	9	2	0	2	0%	0%
986	2	9	2	0	2	0%	0%
597	2	9	2	0	2	0%	0%
1038	2	9	2	0	2	0%	0%
462	2	4	6	1	2.46	0%	0%
752	1	6	5	1	2.46	0%	0%
1037	1	3	7	2	2.76	0%	0%

BURST Group 05: No. of STs = 12 | Predicted Founder = ST-155

					Average	ST Bootstrap	
ST	SLV	DLV	TLV	SAT	Distance	Group	Subgroup
155	11	0	0	0	1	100%	100%
461	4	7	0	0	1.63	5%	10%
280	4	7	0	0	1.63	4%	10%
579	4	7	0	0	1.63	9%	9%
13	4	7	0	0	1.63	15%	10%
786	2	9	0	0	1.81	0%	0%
210	2	9	0	0	1.81	0%	0%
677	1	10	0	0	1.9	0%	0%
250	1	10	0	0	1.9	0%	0%
541	1	10	0	0	1.9	0%	0%
811	1	10	0	0	1.9	0%	0%
1010	1	10	0	0	1.9	0%	0%

BURST Group 06: No. of STs = 12 | Predicted Founder = ST-252

					Average	ST Bootstrap	
ST	SLV	DLV	TLV	SAT	Distance	Group	Subgroup
252	8	3	0	0	1.27	97%	97%
9	5	5	1	0	1.63	34%	44%
495	3	7	1	0	1.81	0%	0%
702	3	7	1	0	1.81	1%	0%
498	3	5	3	0	2	1%	0%
924	3	5	3	0	2	2%	0%
420	3	5	3	0	2	5%	0%
411	2	7	2	0	2	0%	0%
984	1	8	2	0	2.09	0%	0%
163	1	5	4	1	2.45	0%	0%
118	1	4	5	1	2.54	0%	0%
429	1	1	7	2	2.9	0%	0%

BURST Group 07:No. of STs = 9 | Predicted Founder = ST-274

					Average	ST Bootstrap	
ST	SLV	DLV	TLV	SAT	Distance	Group	Subgroup
274	8	0	0	0	1	100%	99%
466	3	5	0	0	1.62	3%	0%
276	3	5	0	0	1.62	3%	0%
209	3	5	0	0	1.62	9%	0%
936	1	7	0	0	1.87	0%	0%
268	1	7	0	0	1.87	0%	0%
546	1	7	0	0	1.87	0%	0%
1068	1	7	0	0	1.87	0%	0%
1043	1	7	0	0	1.87	0%	0%

BURST Group 08: No. of STs = 8 | Predicted Founder = ST-395

					Average	ST Bootstrap	
**ST**	**SLV**	**DLV**	**TLV**	**SAT**	**Distance**	**Group**	**Subgroup**
395	7	0	0	0	1	99%	99%
661	3	4	0	0	1.57	3%	0%
112	3	4	0	0	1.57	3%	0%
407	3	4	0	0	1.57	6%	0%
618	2	5	0	0	1.71	0%	0%
442	2	5	0	0	1.71	0%	0%
632	1	6	0	0	1.85	0%	0%
625	1	6	0	0	1.85	0%	0%

BURST Group 09: No. of STs = 8 | Predicted Founder = ST-179

					Average	ST Bootstrap	
**ST**	**SLV**	**DLV**	**TLV**	**SAT**	**Distance**	**Group**	**Subgroup**
179	5	2	0	0	1.28	90%	77%
180	3	3	1	0	1.71	17%	11%
156	3	3	1	0	1.71	21%	8%
158	2	4	1	0	1.85	0%	0%
353	2	4	1	0	1.85	0%	0%
178	1	5	1	0	2	0%	0%
188	1	3	2	1	2.42	0%	0%
747	1	2	3	1	2.57	0%	0%

BURST Group 10: No. of STs = 8 | Predicted Founder = ST-175

					Average	ST Bootstrap	
**ST**	**SLV**	**DLV**	**TLV**	**SAT**	**Distance**	**Group**	**Subgroup**
175	7	0	0	0	1	99%	98%
619	2	5	0	0	1.71	0%	0%
171	2	5	0	0	1.71	0%	0%
951	2	5	0	0	1.71	0%	0%
159	2	5	0	0	1.71	0%	0%
101	2	5	0	0	1.71	0%	0%
201	2	5	0	0	1.71	0%	0%
228	1	6	0	0	1.85	0%	0%

BURST Group 11: No. of STs = 8 | Predicted Founder = ST-406

					Average	ST Bootstrap	
**ST**	**SLV**	**DLV**	**TLV**	**SAT**	**Distance**	**Group**	**Subgroup**
406	7	0	0	0	1	97%	98%
484	4	3	0	0	1.42	9%	11%
405	4	3	0	0	1.42	5%	12%
536	4	3	0	0	1.42	9%	13%
523	4	3	0	0	1.42	14%	11%
489	2	5	0	0	1.71	0%	0%
547	2	5	0	0	1.71	0%	0%
608	1	6	0	0	1.85	0%	0%

BURST Group 12: No. of STs = 8 | Predicted Founder = ST-835

ST	SLV	DLV	TLV	SAT	Average Distance	ST Bootstrap Group	Subgroup
835	3	3	1	0	1.71	36%	19%
731	3	2	1	1	2	29%	18%
1052	3	2	1	1	2	32%	21%
891	2	3	2	0	2	10%	0%
272	2	1	2	2	2.57	13%	0%
932	1	3	1	2	2.71	0%	0%
838	1	3	1	2	2.71	0%	0%
384	1	1	1	4	3.42	0%	0%

BURST Group 13: No. of STs = 7 | Predicted Founder = ST-560

ST	SLV	DLV	TLV	SAT	Average Distance	ST Bootstrap Group	Subgroup
560	6	0	0	0	1	97%	92%
332	3	3	0	0	1.5	4%	1%
137	3	3	0	0	1.5	6%	1%
340	3	3	0	0	1.5	10%	1%
729	2	4	0	0	1.66	0%	0%
1056	2	4	0	0	1.66	0%	0%
671	1	5	0	0	1.83	0%	0%

BURST Group 14:No. of STs = 7 | Predicted Founder = ST-253

ST	SLV	DLV	TLV	SAT	Average Distance	ST Bootstrap Group	Subgroup
253	4	2	0	0	1.33	73%	52%
540	3	3	0	0	1.5	40%	21%
317	1	3	2	0	2.16	0%	0%
109	1	3	2	0	2.16	0%	0%
828	1	3	2	0	2.16	0%	0%
692	1	2	3	0	2.33	0%	0%
551	1	2	3	0	2.33	0%	0%

BURST Group 15:No. of STs = 7 | Predicted Founder = ST-148

ST	SLV	DLV	TLV	SAT	Average Distance	ST Bootstrap Group	Subgroup
148	6	0	0	0	1	99%	95%
956	2	4	0	0	1.66	0%	0%
140	2	4	0	0	1.66	0%	0%
568	2	4	0	0	1.66	0%	0%
371	2	4	0	0	1.66	0%	0%
682	1	5	0	0	1.83	0%	0%
952	1	5	0	0	1.83	0%	0%

BURST Group 16:No. of STs = 6 | Predicted Founder = ST-132

ST	SLV	DLV	TLV	SAT	Average Distance	ST Bootstrap Group	Subgroup
132	5	0	0	0	1	97%	81%
329	2	3	0	0	1.6	0%	0%
607	2	3	0	0	1.6	0%	0%
665	1	4	0	0	1.8	0%	0%
615	1	4	0	0	1.8	0%	0%
505	1	4	0	0	1.8	0%	0%

BURST Group 17: No. of STs = 6 | Predicted Founder = ST-309

ST	SLV	DLV	TLV	SAT	Average Distance	ST Bootstrap Group	Subgroup
309	5	0	0	0	1	96%	82%
311	2	3	0	0	1.6	0%	0%
658	2	3	0	0	1.6	0%	0%
458	2	3	0	0	1.6	0%	0%
1017	2	3	0	0	1.6	1%	0%
223	1	4	0	0	1.8	0%	0%

BURST Group 18:No. of STs = 6 | Predicted Founder = ST-308

ST	SLV	DLV	TLV	SAT	Average Distance	ST Bootstrap Group	Subgroup
308	5	0	0	0	1	90%	84%
628	3	2	0	0	1.4	6%	5%
1004	3	2	0	0	1.4	6%	6%
345	3	2	0	0	1.4	11%	6%
978	1	4	0	0	1.8	0%	0%
481	1	4	0	0	1.8	0%	0%

BURST Group 19:No. of STs = 6 | Predicted Founder = ST-399

ST	SLV	DLV	TLV	SAT	Average Distance	ST Bootstrap Group	Subgroup
399	4	1	0	0	1.2	73%	49%
656	3	2	0	0	1.4	36%	16%
398	2	3	0	0	1.6	0%	0%
401	1	3	1	0	2	0%	0%
400	1	3	1	0	2	0%	0%
668	1	2	2	0	2.2	0%	0%

BURST Group 20:No. of STs = 6 | Predicted Founder = ST-1058

ST	SLV	DLV	TLV	SAT	Average Distance	ST Bootstrap Group	Subgroup
1058	4	1	0	0	1.2	70%	53%
258	3	2	0	0	1.4	38%	16%
626	2	3	0	0	1.6	1%	0%
734	2	2	1	0	1.8	1%	0%
1055	2	2	1	0	1.8	0%	0%
167	1	2	2	0	2.2	0%	0%

BURST Group 21:No. of STs = 5 | Predicted Founder = ST-620

ST	SLV	DLV	TLV	SAT	Average Distance	ST Bootstrap Group	Subgroup
620	4	0	0	0	1	67%	50%
338	3	1	0	0	1.25	8%	11%
320	3	1	0	0	1.25	12%	10%
753	3	1	0	0	1.25	23%	10%
520	1	3	0	0	1.75	0%	0%

BURST Group 22:No. of STs = 5 | Predicted Founder = ST-298

ST	SLV	DLV	TLV	SAT	Average Distance	ST Bootstrap Group	Subgroup
298	3	1	0	0	1.25	61%	16%
446	2	2	0	0	1.5	17%	0%
691	1	2	1	0	2	0%	0%
539	1	2	1	0	2	0%	0%
650	1	1	2	0	2.25	0%	0%

BURST Group 23:No. of STs = 5 | Predicted Founder = ST-170

ST	SLV	DLV	TLV	SAT	Average Distance	ST Bootstrap Group	Subgroup
170	3	1	0	0	1.25	60%	17%
367	2	2	0	0	1.5	17%	0%
652	1	2	1	0	2	0%	0%
997	1	2	1	0	2	0%	0%
373	1	1	2	0	2.25	0%	0%

BURST Group 24:No. of STs = 5 | Predicted Founder = ST-387

ST	SLV	DLV	TLV	SAT	Average Distance	ST Bootstrap Group	Subgroup
387	3	1	0	0	1.25	62%	16%
181	2	2	0	0	1.5	19%	0%
403	1	3	0	0	1.75	0%	0%
980	1	2	1	0	2	0%	0%
730	1	2	1	0	2	0%	0%

BURST Group 25:No. of STs = 5 | Predicted Founder = ST-282

ST	SLV	DLV	TLV	SAT	Average Distance	ST Bootstrap Group	Subgroup
282	4	0	0	0	1	89%	52%
444	2	2	0	0	1.5	1%	0%
278	2	2	0	0	1.5	2%	0%
959	1	3	0	0	1.75	0%	0%
410	1	3	0	0	1.75	0%	0%

BURST Group 26:No. of STs = 5 | Predicted Founder = Multiple

ST	SLV	DLV	TLV	SAT	Average Distance	ST Bootstrap Group	Subgroup
153	2	2	0	0	1.5	26%	0%
219	2	2	0	0	1.5	20%	0%
887	2	1	1	0	1.75	22%	0%
583	1	2	1	0	2	0%	0%
41	1	1	2	0	2.25	0%	0%

BURST Group 27:No. of STs = 5 | Predicted Founder = ST-245

ST	SLV	DLV	TLV	SAT	Average Distance	ST Bootstrap Group	Subgroup
245	4	0	0	0	1	79%	46%
142	2	2	0	0	1.5	1%	0%
740	2	2	0	0	1.5	3%	0%
580	2	2	0	0	1.5	2%	0%
561	2	2	0	0	1.5	5%	0%

BURST Group 28:No. of STs = 5 | Predicted Founder = ST-514

ST	SLV	DLV	TLV	SAT	Average Distance	ST Bootstrap Group	Subgroup
514	3	1	0	0	1.25	60%	14%
482	2	2	0	0	1.5	19%	0%
133	1	2	1	0	2	0%	0%
556	1	2	1	0	2	0%	0%
513	1	1	2	0	2.25	0%	0%

BURST Group 29:No. of STs = 4 | Predicted Founder = ST-313

ST	SLV	DLV	TLV	SAT	Average Distance	ST Bootstrap Group	Subgroup
313	3	0	0	0	1	73%	14%
678	1	2	0	0	1.66	0%	0%
648	1	2	0	0	1.66	0%	0%
174	1	2	0	0	1.66	0%	0%

BURST Group 30:No. of STs = 4 | Predicted Founder = ST-277

ST	SLV	DLV	TLV	SAT	Average Distance	ST Bootstrap Group	Subgroup
277	3	0	0	0	1	61%	13%
659	2	1	0	0	1.33	5%	0%
758	2	1	0	0	1.33	11%	0%
364	1	2	0	0	1.66	0%	0%

BURST Group 31:No. of STs = 4 | Predicted Founder = ST-1025

ST	SLV	DLV	TLV	SAT	Average Distance	ST Bootstrap Group	Subgroup
1025	3	0	0	0	1	60%	13%
645	2	1	0	0	1.33	6%	0%
256	2	1	0	0	1.33	10%	0%
968	1	2	0	0	1.66	0%	0%

BURST Group 32:No. of STs = 4 | Predicted Founder = ST-233

ST	SLV	DLV	TLV	SAT	Average Distance	ST Bootstrap Group	Subgroup
233	3	0	0	0	1	75%	13%
629	1	2	0	0	1.66	0%	0%
743	1	2	0	0	1.66	0%	0%
742	1	2	0	0	1.66	0%	0%

BURST Group 33:No. of STs = 4 | Predicted Founder = ST-152

ST	SLV	DLV	TLV	SAT	Average Distance	ST Bootstrap Group	Subgroup
152	3	0	0	0	1	74%	12%
185	1	2	0	0	1.66	0%	0%
953	1	2	0	0	1.66	0%	0%
749	1	2	0	0	1.66	0%	0%

BURST Group 34:No. of STs = 4 | Predicted Founder = ST-497

ST	SLV	DLV	TLV	SAT	Average Distance	ST Bootstrap Group	Subgroup
497	3	0	0	0	1	58%	13%
602	2	1	0	0	1.33	5%	0%
576	2	1	0	0	1.33	12%	0%
544	1	2	0	0	1.66	0%	0%

BURST Group 35:No. of STs = 4 | Predicted Founder = ST-217

ST	SLV	DLV	TLV	SAT	Average Distance	ST Bootstrap Group	Subgroup
217	3	0	0	0	1	71%	11%
950	1	2	0	0	1.66	0%	0%
417	1	2	0	0	1.66	0%	0%
14	1	2	0	0	1.66	0%	0%

BURST Group 36:No. of STs = 4 | Predicted Founder = ST-571

ST	SLV	DLV	TLV	SAT	Average Distance	ST Bootstrap Group	Subgroup
571	2	1	0	0	1.33	29%	0%
1012	2	1	0	0	1.33	25%	0%
938	1	1	1	0	2	0%	0%
1013	1	1	1	0	2	0%	0%

BURST Group 37:No. of STs = 4 | Predicted Founder = ST-385

ST	SLV	DLV	TLV	SAT	Average Distance	ST Bootstrap Group	Subgroup
385	3	0	0	0	1	58%	14%
920	2	1	0	0	1.33	3%	0%
1036	2	1	0	0	1.33	12%	0%
443	1	2	0	0	1.66	0%	0%

BURST Group 38:No. of STs = 4 | Predicted Founder = ST-569

ST	SLV	DLV	TLV	SAT	Average Distance	ST Bootstrap Group	Subgroup
569	3	0	0	0	1	73%	12%
432	1	2	0	0	1.66	0%	0%
529	1	2	0	0	1.66	0%	0%
848	1	2	0	0	1.66	0%	0%

BURST Group 39:No. of STs = 4 | Predicted Founder = ST-782

ST	SLV	DLV	TLV	SAT	Average Distance	ST Bootstrap Group	Subgroup
782	3	0	0	0	1	73%	12%
785	1	2	0	0	1.66	0%	0%
784	1	2	0	0	1.66	0%	0%
783	1	2	0	0	1.66	0%	0%

BURST Group 40:No. of STs = 4 | Predicted Founder = ST-238

ST	SLV	DLV	TLV	SAT	Average Distance	ST Bootstrap Group	Subgroup
238	3	0	0	0	1	58%	12%
243	2	1	0	0	1.33	5%	0%
237	2	1	0	0	1.33	11%	0%
1001	1	2	0	0	1.66	0%	0%

BURST Group 41:No. of STs = 4 | Predicted Founder = ST-598

ST	SLV	DLV	TLV	SAT	Average Distance	ST Bootstrap Group	Subgroup
598	3	0	0	0	1	59%	15%
896	2	1	0	0	1.33	4%	0%
847	2	1	0	0	1.33	10%	0%
226	1	2	0	0	1.66	0%	0%

BURST Group 42:No. of STs = 4 | Predicted Founder = ST-360

ST	SLV	DLV	TLV	SAT	Average Distance	ST Bootstrap Group	Subgroup
360	2	1	0	0	1.33	26%	0%
861	2	1	0	0	1.33	29%	0%
864	1	1	1	0	2	0%	0%
862	1	1	1	0	2	0%	0%

BURST Group 43:No. of STs = 3 | Predicted Founder = ST-699

ST	SLV	DLV	TLV	SAT	Average Distance	ST Bootstrap Group	Subgroup
699	2	0	0	0	1	33%	0%
944	1	1	0	0	1.5	0%	0%
549	1	1	0	0	1.5	0%	0%

BURST Group 44:No. of STs = 3 | Predicted Founder = ST-273

ST	SLV	DLV	TLV	SAT	Average Distance	ST Bootstrap Group	Subgroup
273	2	0	0	0	1	31%	0%
695	1	1	0	0	1.5	0%	0%
510	1	1	0	0	1.5	0%	0%

BURST Group 45:No. of STs = 3 | Predicted Founder = ST-348

ST	SLV	DLV	TLV	SAT	Average Distance	ST Bootstrap Group	Subgroup
348	2	0	0	0	1	33%	0%
331	1	1	0	0	1.5	0%	0%
416	1	1	0	0	1.5	0%	0%

BURST Group 46:No. of STs = 3 | Predicted Founder = ST-676

ST	SLV	DLV	TLV	SAT	Average Distance	ST Bootstrap Group	Subgroup
676	2	0	0	0	1	33%	0%
128	1	1	0	0	1.5	0%	0%
450	1	1	0	0	1.5	0%	0%

BURST Group 47:No. of STs = 3 | Predicted Founder = ST-667

ST	SLV	DLV	TLV	SAT	Average Distance	ST Bootstrap Group	Subgroup
667	2	0	0	0	1	32%	0%
468	1	1	0	0	1.5	0%	0%
502	1	1	0	0	1.5	0%	0%

BURST Group 48:No. of STs = 3 | Predicted Founder = ST-254

ST	SLV	DLV	TLV	SAT	Average Distance	ST Bootstrap Group	Subgroup
254	2	0	0	0	1	31%	0%
666	1	1	0	0	1.5	0%	0%
1041	1	1	0	0	1.5	0%	0%

BURST Group 49:No. of STs = 3 | Predicted Founder = ST-709

ST	SLV	DLV	TLV	SAT	Average Distance	ST Bootstrap Group	Subgroup
709	2	0	0	0	1	31%	0%
655	1	1	0	0	1.5	0%	0%
738	1	1	0	0	1.5	0%	0%

BURST Group 50:No. of STs = 3 | Predicted Founder = ST-964

ST	SLV	DLV	TLV	SAT	Average Distance	ST Bootstrap Group	Subgroup
964	2	0	0	0	1	31%	0%
654	1	1	0	0	1.5	0%	0%
741	1	1	0	0	1.5	0%	0%

BURST Group 51:No. of STs = 3 | Predicted Founder = Multiple

ST	SLV	DLV	TLV	SAT	Average Distance	ST Bootstrap Group	Subgroup
638	2	0	0	0	1	6%	0%
797	2	0	0	0	1	8%	0%
378	2	0	0	0	1	18%	0%

BURST Group 52:No. of STs = 3 | Predicted Founder = ST-242

ST	SLV	DLV	TLV	SAT	Average Distance	ST Bootstrap Group	Subgroup
242	2	0	0	0	1	31%	0%
996	1	1	0	0	1.5	0%	0%
456	1	1	0	0	1.5	0%	0%

BURST Group 53:No. of STs = 3 | Predicted Founder = ST-195

ST	SLV	DLV	TLV	SAT	Average Distance	ST Bootstrap Group	Subgroup
195	2	0	0	0	1	29%	0%
977	1	1	0	0	1.5	0%	0%
349	1	1	0	0	1.5	0%	0%

BURST Group 54:No. of STs = 3 | Predicted Founder = ST-164

ST	SLV	DLV	TLV	SAT	Average Distance	ST Bootstrap Group	Subgroup
164	2	0	0	0	1	28%	0%
193	1	1	0	0	1.5	0%	0%
512	1	1	0	0	1.5	0%	0%

BURST Group 55:No. of STs = 3 | Predicted Founder = ST-192

ST	SLV	DLV	TLV	SAT	Average Distance	ST Bootstrap Group	Subgroup
192	2	0	0	0	1	30%	0%
165	1	1	0	0	1.5	0%	0%
220	1	1	0	0	1.5	0%	0%

BURST Group 56:No. of STs = 3 | Predicted Founder = ST-139

ST	SLV	DLV	TLV	SAT	Average Distance	ST Bootstrap Group	Subgroup
139	2	0	0	0	1	31%	0%
115	1	1	0	0	1.5	0%	0%
452	1	1	0	0	1.5	0%	0%

BURST Group 57:No. of STs = 3 | Predicted Founder = ST-365

ST	SLV	DLV	TLV	SAT	Average Distance	ST Bootstrap Group	Subgroup
365	2	0	0	0	1	33%	0%
922	1	1	0	0	1.5	0%	0%
522	1	1	0	0	1.5	0%	0%

BURST Group 58:No. of STs = 3 | Predicted Founder = ST-485

ST	SLV	DLV	TLV	SAT	Average Distance	ST Bootstrap Group	Subgroup
485	2	0	0	0	1	30%	0%
855	1	1	0	0	1.5	0%	0%
1033	1	1	0	0	1.5	0%	0%

BURST Group 59:No. of STs = 3 | Predicted Founder = ST-389

ST	SLV	DLV	TLV	SAT	Average Distance	ST Bootstrap Group	Subgroup
389	2	0	0	0	1	31%	0%
121	1	1	0	0	1.5	0%	0%
424	1	1	0	0	1.5	0%	0%

BURST Group 60:No. of STs = 3 | Predicted Founder = ST-108

ST	SLV	DLV	TLV	SAT	Average Distance	ST Bootstrap Group	Subgroup
108	2	0	0	0	1	30%	0%
425	1	1	0	0	1.5	0%	0%
581	1	1	0	0	1.5	0%	0%

BURST Group 61:No. of STs = 3 | Predicted Founder = ST-260

ST	SLV	DLV	TLV	SAT	Average Distance	ST Bootstrap Group	Subgroup
260	2	0	0	0	1	31%	0%
264	1	1	0	0	1.5	0%	0%
503	1	1	0	0	1.5	0%	0%

BURST Group 62:No. of STs = 3 | Predicted Founder = ST-236

ST	SLV	DLV	TLV	SAT	Average Distance	ST Bootstrap Group	Subgroup
236	2	0	0	0	1	31%	0%
240	1	1	0	0	1.5	0%	0%
239	1	1	0	0	1.5	0%	0%

BURST Group 63:No. of STs = 3 | Predicted Founder = ST-231

ST	SLV	DLV	TLV	SAT	Average Distance	ST Bootstrap Group	Subgroup
231	2	0	0	0	1	34%	0%
376	1	1	0	0	1.5	0%	0%
370	1	1	0	0	1.5	0%	0%

BURST Group 64:No. of STs = 3 | Predicted Founder = Multiple

ST	SLV	DLV	TLV	SAT	Average Distance	ST Bootstrap Group	Subgroup
525	2	0	0	0	1	8%	0%
354	2	0	0	0	1	9%	0%
350	2	0	0	0	1	13%	0%

BURST Group 65:No. of STs = 3 | Predicted Founder = ST-377

ST	SLV	DLV	TLV	SAT	Average Distance	ST Bootstrap Group	Subgroup
377	2	0	0	0	1	6%	0%
1047	2	0	0	0	1	8%	0%
1028	2	0	0	0	1	14%	0%

BURST Group 66:No. of STs = 3 | Predicted Founder = ST-368

ST	SLV	DLV	TLV	SAT	Average Distance	ST Bootstrap Group	Subgroup
368	2	0	0	0	1	31%	0%
372	1	1	0	0	1.5	0%	0%
366	1	1	0	0	1.5	0%	0%

BURST Group 67:No. of STs = 2 | Predicted Founder = None

ST	SLV	DLV	TLV	SAT	Distance
327	1	0	0	0	1
325	1	0	0	0	1

BURST Group 68:No. of STs = 2 | Predicted Founder = None

ST	SLV	DLV	TLV	SAT	Distance
685	1	0	0	0	1
684	1	0	0	0	1

BURST Group 69:No. of STs = 2 | Predicted Founder = None

ST	SLV	DLV	TLV	SAT	Distance
319	1	0	0	0	1
486	1	0	0	0	1

BURST Group 70:No. of STs = 2 | Predicted Founder = None

ST	SLV	DLV	TLV	SAT	Distance
316	1	0	0	0	1
507	1	0	0	0	1

BURST Group 71:No. of STs = 2 | Predicted Founder = None

ST	SLV	DLV	TLV	SAT	Distance
675	1	0	0	0	1
1008	1	0	0	0	1

BURST Group 72:No. of STs = 2 | Predicted Founder = None

ST	SLV	DLV	TLV	SAT	Distance
312	1	0	0	0	1
279	1	0	0	0	1

BURST Group 73:No. of STs = 2 | Predicted Founder = None

ST	SLV	DLV	TLV	SAT	Distance
310	1	0	0	0	1
306	1	0	0	0	1

BURST Group 74:No. of STs = 2 | Predicted Founder = None

ST	SLV	DLV	TLV	SAT	Distance
307	1	0	0	0	1
1027	1	0	0	0	1

BURST Group 75:No. of STs = 2 | Predicted Founder = None

ST	SLV	DLV	TLV	SAT	Distance
663	1	0	0	0	1
131	1	0	0	0	1

BURST Group 76:No. of STs = 2 | Predicted Founder = None

ST	SLV	DLV	TLV	SAT	Distance
300	1	0	0	0	1
491	1	0	0	0	1

BURST Group 77:No. of STs = 2 | Predicted Founder = None

ST	SLV	DLV	TLV	SAT	Distance
642	1	0	0	0	1
211	1	0	0	0	1

BURST Group 78:No. of STs = 2 | Predicted Founder = None

ST	SLV	DLV	TLV	SAT	Distance
639	1	0	0	0	1
853	1	0	0	0	1

BURST Group 79:No. of STs = 2 | Predicted Founder = None

ST	SLV	DLV	TLV	SAT	Distance
635	1	0	0	0	1
480	1	0	0	0	1

BURST Group 80:No. of STs = 2 | Predicted Founder = None

ST	SLV	DLV	TLV	SAT	Distance
633	1	0	0	0	1
1066	1	0	0	0	1

BURST Group 81:No. of STs = 2 | Predicted Founder = None

ST	SLV	DLV	TLV	SAT	Distance
198	1	0	0	0	1
1048	1	0	0	0	1

BURST Group 82:No. of STs = 2 | Predicted Founder = None

ST	SLV	DLV	TLV	SAT	Distance
197	1	0	0	0	1
125	1	0	0	0	1

BURST Group 83:No. of STs = 2 | Predicted Founder = None

ST	SLV	DLV	TLV	SAT	Distance
190	1	0	0	0	1
566	1	0	0	0	1

BURST Group 84:No. of STs = 2 | Predicted Founder = None

ST	SLV	DLV	TLV	SAT	Distance
981	1	0	0	0	1
880	1	0	0	0	1

BURST Group 85:No. of STs = 2 | Predicted Founder = None

ST	SLV	DLV	TLV	SAT	Distance
184	1	0	0	0	1
160	1	0	0	0	1

BURST Group 86:No. of STs = 2 | Predicted Founder = None

ST	SLV	DLV	TLV	SAT	Distance
974	1	0	0	0	1
413	1	0	0	0	1

BURST Group 87:No. of STs = 2 | Predicted Founder = None

ST	SLV	DLV	TLV	SAT	Distance
611	1	0	0	0	1
767	1	0	0	0	1

BURST Group 88:No. of STs = 2 | Predicted Founder = None

ST	SLV	DLV	TLV	SAT	Distance
177	1	0	0	0	1
176	1	0	0	0	1

BURST Group 89:No. of STs = 2 | Predicted Founder = None

ST	SLV	DLV	TLV	SAT	Distance
173	1	0	0	0	1
1031	1	0	0	0	1

BURST Group 90:No. of STs = 2 | Predicted Founder = None

ST	SLV	DLV	TLV	SAT	Distance
965	1	0	0	0	1
528	1	0	0	0	1

BURST Group 91:No. of STs = 2 | Predicted Founder = None

ST	SLV	DLV	TLV	SAT	Distance
564	1	0	0	0	1
604	1	0	0	0	1

BURST Group 92:No. of STs = 2 | Predicted Founder = None

ST	SLV	DLV	TLV	SAT	Distance
601	1	0	0	0	1
53	1	0	0	0	1

BURST Group 93:No. of STs = 2 | Predicted Founder = None

ST	SLV	DLV	TLV	SAT	Distance
954	1	0	0	0	1
281	1	0	0	0	1

BURST Group 94:No. of STs = 2 | Predicted Founder = None

ST	SLV	DLV	TLV	SAT	Distance
931	1	0	0	0	1
881	1	0	0	0	1

BURST Group 95:No. of STs = 2 | Predicted Founder = None

ST	SLV	DLV	TLV	SAT	Distance
701	1	0	0	0	1
493	1	0	0	0	1

BURST Group 96:No. of STs = 2 | Predicted Founder = None

ST	SLV	DLV	TLV	SAT	Distance
490	1	0	0	0	1
535	1	0	0	0	1

BURST Group 97:No. of STs = 2 | Predicted Founder = None

ST	SLV	DLV	TLV	SAT	Distance
487	1	0	0	0	1
737	1	0	0	0	1

BURST Group 98:No. of STs = 2 | Predicted Founder = None

ST	SLV	DLV	TLV	SAT	Distance
917	1	0	0	0	1
214	1	0	0	0	1

BURST Group 99:No. of STs = 2 | Predicted Founder = None

ST	SLV	DLV	TLV	SAT	Distance
475	1	0	0	0	1
527	1	0	0	0	1

BURST Group 100:No. of STs = 2 | Predicted Founder = None

ST	SLV	DLV	TLV	SAT	Distance
110	1	0	0	0	1
363	1	0	0	0	1

BURST Group 101:No. of STs = 2 | Predicted Founder = None

ST	SLV	DLV	TLV	SAT	Distance
455	1	0	0	0	1
578	1	0	0	0	1

BURST Group 102:No. of STs = 2 | Predicted Founder = None

ST	SLV	DLV	TLV	SAT	Distance
448	1	0	0	0	1
447	1	0	0	0	1

BURST Group 103:No. of STs = 2 | Predicted Founder = None

ST	SLV	DLV	TLV	SAT	Distance
440	1	0	0	0	1
267	1	0	0	0	1

BURST Group 104:No. of STs = 2 | Predicted Founder = None

ST	SLV	DLV	TLV	SAT	Distance
439	1	0	0	0	1
515	1	0	0	0	1

BURST Group 105:No. of STs = 2 | Predicted Founder = None

ST	SLV	DLV	TLV	SAT	Distance
799	1	0	0	0	1
800	1	0	0	0	1

BURST Group 106:No. of STs = 2 | Predicted Founder = None

ST	SLV	DLV	TLV	SAT	Distance
798	1	0	0	0	1
736	1	0	0	0	1

BURST Group 107:No. of STs = 2 | Predicted Founder = None

ST	SLV	DLV	TLV	SAT	Distance
434	1	0	0	0	1
840	1	0	0	0	1

BURST Group 108:No. of STs = 2 | Predicted Founder = None

ST	SLV	DLV	TLV	SAT	Distance
433	1	0	0	0	1
552	1	0	0	0	1

BURST Group 109:No. of STs = 2 | Predicted Founder = None

ST	SLV	DLV	TLV	SAT	Distance
428	1	0	0	0	1
427	1	0	0	0	1

BURST Group 110:No. of STs = 2 | Predicted Founder = None

ST	SLV	DLV	TLV	SAT	Distance
788	1	0	0	0	1
787	1	0	0	0	1

BURST Group 111:No. of STs = 2 | Predicted Founder = None

ST	SLV	DLV	TLV	SAT	Distance
257	1	0	0	0	1
780	1	0	0	0	1

BURST Group 112:No. of STs = 2 | Predicted Founder = None

ST	SLV	DLV	TLV	SAT	Distance
262	1	0	0	0	1
774	1	0	0	0	1

BURST Group 113:No. of STs = 2 | Predicted Founder = None

ST	SLV	DLV	TLV	SAT	Distance
761	1	0	0	0	1
591	1	0	0	0	1

BURST Group 114:No. of STs = 2 | Predicted Founder = None

ST	SLV	DLV	TLV	SAT	Distance
756	1	0	0	0	1
712	1	0	0	0	1

BURST Group 115:No. of STs = 2 | Predicted Founder = None

ST	SLV	DLV	TLV	SAT	Distance
755	1	0	0	0	1
751	1	0	0	0	1

BURST Group 116:No. of STs = 2 | Predicted Founder = None

ST	SLV	DLV	TLV	SAT	Distance
234	1	0	0	0	1
754	1	0	0	0	1

BURST Group 117:No. of STs = 2 | Predicted Founder = None

ST	SLV	DLV	TLV	SAT	Distance
746	1	0	0	0	1
357	1	0	0	0	1

BURST Group 118:No. of STs = 2 | Predicted Founder = None

ST	SLV	DLV	TLV	SAT	Distance
733	1	0	0	0	1
392	1	0	0	0	1

BURST Group 119:No. of STs = 2 | Predicted Founder = None

ST	SLV	DLV	TLV	SAT	Distance
532	1	0	0	0	1
297	1	0	0	0	1

BURST Group 120:No. of STs = 2 | Predicted Founder = None

ST	SLV	DLV	TLV	SAT	Distance
296	1	0	0	0	1
538	1	0	0	0	1

BURST Group 121:No. of STs = 2 | Predicted Founder = None

ST	SLV	DLV	TLV	SAT	Distance
288	1	0	0	0	1
287	1	0	0	0	1

BURST Group 122:No. of STs = 2 | Predicted Founder = None

ST	SLV	DLV	TLV	SAT	Distance
719	1	0	0	0	1
224	1	0	0	0	1

BURST Group 123:No. of STs = 2 | Predicted Founder = None

ST	SLV	DLV	TLV	SAT	Distance
266	1	0	0	0	1
873	1	0	0	0	1

BURST Group 124:No. of STs = 2 | Predicted Founder = None

ST	SLV	DLV	TLV	SAT	Distance
265	1	0	0	0	1
248	1	0	0	0	1

BURST Group 125:No. of STs = 2 | Predicted Founder = None

ST	SLV	DLV	TLV	SAT	Distance
261	1	0	0	0	1
810	1	0	0	0	1

BURST Group 126:No. of STs = 2 | Predicted Founder = None

ST	SLV	DLV	TLV	SAT	Distance
596	1	0	0	0	1
871	1	0	0	0	1

BURST Group 127:No. of STs = 2 | Predicted Founder = None

ST	SLV	DLV	TLV	SAT	Distance
575	1	0	0	0	1
1003	1	0	0	0	1

BURST Group 128:No. of STs = 2 | Predicted Founder = None

ST	SLV	DLV	TLV	SAT	Distance
555	1	0	0	0	1
573	1	0	0	0	1

BURST Group 129:No. of STs = 2 | Predicted Founder = None

ST	SLV	DLV	TLV	SAT	Distance
554	1	0	0	0	1
804	1	0	0	0	1

BURST Group 130:No. of STs = 2 | Predicted Founder = None

ST	SLV	DLV	TLV	SAT	Distance
543	1	0	0	0	1
1054	1	0	0	0	1

BURST Group 131:No. of STs = 2 | Predicted Founder = None

ST	SLV	DLV	TLV	SAT	Distance
889	1	0	0	0	1
884	1	0	0	0	1

BURST Group 132:No. of STs = 2 | Predicted Founder = None

ST	SLV	DLV	TLV	SAT	Distance
859	1	0	0	0	1
858	1	0	0	0	1

BURST Group 133:No. of STs = 2 | Predicted Founder = None

ST	SLV	DLV	TLV	SAT	Distance
1067	1	0	0	0	1
1009	1	0	0	0	1

BURST Group 134:No. of STs = 2 | Predicted Founder = None

ST	SLV	DLV	TLV	SAT	Distance
843	1	0	0	0	1
3	1	0	0	0	1

BURST Group 135:No. of STs = 2 | Predicted Founder = None

ST	SLV	DLV	TLV	SAT	Distance
397	1	0	0	0	1
396	1	0	0	0	1

BURST Group 136:No. of STs = 2 | Predicted Founder = None

ST	SLV	DLV	TLV	SAT	Distance
386	1	0	0	0	1
394	1	0	0	0	1

BURST Group 137:No. of STs = 2 | Predicted Founder = None

ST	SLV	DLV	TLV	SAT	Distance
52	1	0	0	0	1
45	1	0	0	0	1

BURST Group 138:No. of STs = 2 | Predicted Founder = None

ST	SLV	DLV	TLV	SAT	Distance
381	1	0	0	0	1
343	1	0	0	0	1

BURST Group 139:No. of STs = 2 | Predicted Founder = None

ST	SLV	DLV	TLV	SAT	Distance
46	1	0	0	0	1
38	1	0	0	0	1

BURST Group 140:No. of STs = 2 | Predicted Founder = None

ST	SLV	DLV	TLV	SAT	Distance
347	1	0	0	0	1
43	1	0	0	0	1

BURST Group 141:No. of STs = 2 | Predicted Founder = None

ST	SLV	DLV	TLV	SAT	Distance
1021	1	0	0	0	1
341	1	0	0	0	1

BURST Group 142:No. of STs = 2 | Predicted Founder = None

ST	SLV	DLV	TLV	SAT	Distance
39	1	0	0	0	1
31	1	0	0	0	1

BURST Group 143:No. of STs = 2 | Predicted Founder = None

ST	SLV	DLV	TLV	SAT	Distance
806	1	0	0	0	1
805	1	0	0	0	1

BURST Group 144:No. of STs = 2 | Predicted Founder = None

ST	SLV	DLV	TLV	SAT	Distance
801	1	0	0	0	1
4	1	0	0	0	1

BURST Group 145:No. of STs = 2 | Predicted Founder = None

ST	SLV	DLV	TLV	SAT	Distance
356	1	0	0	0	1
355	1	0	0	0	1

Singletons: No. of STs = 514

1	44	79	123	203	295	393	477	559
2	47	80	124	204	299	402	478	563
5	48	81	126	205	301	404	479	565
6	49	82	127	206	302	408	483	567
7	50	83	130	207	303	409	488	570
8	51	84	134	208	305	415	492	572
10	54	85	135	212	314	419	494	574
11	55	86	136	213	315	422	496	582
12	56	87	138	215	324	423	499	584
15	57	88	143	216	326	426	500	585
16	58	89	144	218	328	430	501	586
18	59	90	145	221	330	431	504	587
19	60	91	147	222	333	435	506	588
20	61	92	149	225	335	436	511	590
21	62	93	150	229	337	437	516	592
22	63	94	151	246	344	438	517	599
23	64	95	154	249	346	451	518	600
24	65	96	161	251	351	453	519	603
25	66	97	162	255	352	454	521	605
26	67	98	166	259	358	459	524	606
28	68	99	168	263	359	460	526	609
29	69	100	169	270	361	463	530	610
30	70	103	172	271	362	464	531	612
32	71	104	183	275	369	465	537	613
33	72	105	186	285	375	467	542	614
34	73	106	189	286	379	469	545	616
35	74	107	191	289	382	470	548	617
36	75	114	194	290	383	472	550	621
37	76	116	196	291	388	473	553	623
40	77	117	199	292	390	474	557	624
42	78	122	200	293	391	476	558	627

Singletons (continued)

631	748	841	913	1000
634	750	842	915	1002
637	757	844	916	1005
640	759	846	918	1006
643	760	849	919	1007
644	762	850	921	1011
646	763	851	923	1014
647	764	852	925	1016
649	765	854	926	1018
657	768	856	927	1019
662	769	857	928	1020
664	770	860	929	1022
670	771	863	930	1023
672	773	865	933	1024
673	776	867	935	1026
674	777	868	939	1029
679	778	869	940	1030
689	779	870	941	1032
690	781	872	942	1034
693	789	874	943	1035
694	790	875	945	1039
697	792	876	946	1040
698	793	877	947	1042
703	794	878	948	1044
704	795	879	949	1045
705	796	882	955	1046
706	802	885	957	1049
707	807	888	960	1050
708	809	890	961	1051
710	812	892	962	1053
711	813	893	963	1057
713	815	894	967	1059
714	816	895	969	1060
715	817	897	971	1061
716	818	898	972	1062
717	819	899	975	1063
718	820	900	979	1064
720	821	901	982	1069
721	822	902	983	1070
722	823	903	985	
723	825	904	987	
724	826	905	988	
725	827	906	991	
726	829	907	992	
727	830	908	993	
728	831	909	994	
732	832	910	995	
735	836	911	998	
744	837	912	999	
